# Promoting free flow in the networks: Reimagining the body in early modern Suzhou

**DOI:** 10.1177/0073275317709406

**Published:** 2017-07-10

**Authors:** Volker Scheid

**Affiliations:** EAST*medicine* Research Group, Faculty of Science and Technology, University of Westminster, UK

**Keywords:** China, Chinese science, practices of knowing, epistemology, medicine, Chinese medicine, global early modern

## Abstract

The history of Chinese medicine is still widely imagined in terms dictated by the discourse of modernity, that is as ‘traditional’ and ‘Chinese.’ And yet, so as to be intelligible to us moderns, it must simultaneously be framed through categories that make it comparable somehow to the ‘West’ and the ‘modern’ from which it is said to be essentially different. This is accomplished, for instance, by viewing Chinese medicine as fundamentally shaped by cosmological thinking, as focusing on process rather than matter, and as forever hampered by attachments to the past even when it tries to innovate. At the same time, it is described as pursuing its objectives in ways that make sense in ‘our’ terms, too, such as the goal of creating physiological homeostasis through methods of supplementation and drainage. In this paper, I seek to move beyond this kind of analysis through a two-pronged approach. First, by focusing on the concept of *tong* 通 - a character that calls forth images of free flow, connectivity, relatedness and understanding - I foreground an important aspect of Chinese medical thinking and practice that has virtually been ignored by Western historians of medicine and science. Second, by exploring how the influential physician Ye Tianshi 葉天士 (1664–1746) employed *tong* to advance medical thinking and practice at a crucial moment of change in the history of Chinese medicine, I demonstrate that physicians in early modern China moved towards new understandings of the body readily intelligible by modern biomedical anatomy. I argue that this mode of analysis allows us to transcend the limitations inherent in the current historiography of Chinese medicine: because it allows for comparison to emerge from our subject matter rather than imposing our imaginaries onto it in advance.

For us moderns, it is difficult to think about Chinese medicine and science without making an obligatory detour via biomedicine and (Western) science. Governmental and supra-governmental discourse, the self-identification of Chinese medicine practitioners, and ethnographic and historical scholarship are all habitually structured around comparisons between China and the West, the traditional and the modern, and the local and the universal. These tendencies persist despite our awareness that the proclaimed universality of science and biomedicine is, in fact, always locally constituted, and despite the increasing range of studies that challenge modernist essentialisms by drawing attention to the historical plurality and heterogeneity of Chinese medicine.

The binary perspective that structures modernity is notably salient in depictions of how Chinese medicine imagined the body, especially in publications that address themselves to non-specialists and, therefore, have the greatest influence on generalist historians of science. In such writings, the Western medical focus on anatomy and structure (widely traced in its emergence to ancient Greece) is invariably contrasted with “the predominantly functional discourse of [Chinese] classical medicine.”^1^ This functional discourse, furthermore, is depicted as being rooted “in cosmological models of how the physical and physiological world worked [that] remained essentially unchanged” throughout Chinese medicine’s long history.^2^ In contrast to biomedicine’s focus on specific disease causes, the supposed “holistic character” of Chinese medicine leads it to view illness “as a loss of harmony in the body’s operations” that should be restored by restoring functional flow and rebalancing *yin* and *yang*.^3,4^

Historical exceptions to these generalizations are then depicted as anomalies. Perhaps the most commonly discussed cases are the anatomical studies of the early nineteenth century physician Wang Qingren 王清任 (1768–1831). In his widely read (and widely critiqued) book *Correcting Errors Among Physicians* (*Yilin gaicuo* 醫林改錯), Wang rejected the depiction of bodily organs in classical texts as mostly wrong and proceeded to correct them on the basis of his personal examination of human corpses. This elicits two different, though ultimately similar, responses among modern historians. One portrays him as “one of the few Chinese to take anatomy seriously,” an outlier whose strange interests can at least partially be explained by the fact that he “seems to have had unwitting access to an indirect transmission of Jesuit anatomy.”^5^ The second portrays Wang as embodying the sprouts of an indigenous turn towards medical modernization, as someone who “called his own work just a beginning” and “could not have suspected that only a few years after his death, the discoveries of medical researchers and practitioners from a foreign civilization, who had already pursued his call for centuries, would raise healing in China to a completely new plane.”^6^

However, if one is willing to abandon, even momentarily, the modernist biases that generate these binaries, a quite different picture begins to take shape. First, the image of Chinese medicine as being concerned with holism, cosmological process, harmony, and flow, rather than anatomical structure, is revealed to be a relatively recent construct. It emerged in the 1920s during heated polemics between advocates of Chinese and Western medicine in China, when Chinese medicine physicians made the strategic decision to no longer defend the anatomical knowledge produced by their own tradition. Instead, they argued that the essential and enduring focus of Chinese medicine was “*qi* transformation” (*qihua* 氣化), a concept that itself was a newly fashioned hybrid of classical medical cosmology and the physics associated with steam engine technology.^7^

Second, before the contest with Western biomedicine compelled them to choose between structure and function, Chinese physicians had continuously struggled to define the material bodily foundations of *qi* flow and transformation without seeing anatomy and process as opposed to each other. Wang Qingren himself, for instance, employed the knowledge he had gained by way of his anatomical studies to compose a range of new medicinal formulas that are still widely used today to order the flow of blood and *qi*.^8^ His anatomical interests, furthermore, were by no means outlandish, even if his methods of advancing them were not mainstream. Beginning in the middle of the sixteenth century, physicians in China’s Yangzi river delta – an area known in Chinese as Jiangnan 江南 – had embarked on a broad reexamination of bodily structure and its relation to bodily function and therapeutics. They did so in conversation with earlier authors and texts, not only the early classics but specifically also those from another period of innovation in the twelfth and thirteenth centuries, all of which had had similar concerns that were not triggered, in any significant way, by the influx of Western anatomical knowledge.^9^

In this paper, I examine these transformations by analyzing how the physician Ye Tianshi 葉天士 (1664–1746) applied the philosophical/anatomical/physiological concept of *tong* 通 to the bodily structures known in Chinese medicine as the “network vessels” (*luomai* 絡脈), often simply shortened to “networks” (*luo* 絡). As one of the most famous and influential physicians in the history of Chinese medicine, Ye Tianshi constitutes an ideal focus for the investigation I have in mind. The networks, likewise, are well known to historians of Chinese medicine, but changes in how they were imagined and deployed in clinical practice have not yet been examined. And while *tong* is one of the central concepts in Chinese thinking and culture, its role in and importance to Chinese medicine have virtually gone unnoticed to date.

I will show how Ye Tianshi exploited the potential offered to him by the ancient concept of *tong* 通 as well as by newly emergent ideas about the nature of the networks, using them to engage with and productively resolve the distinctive historical problematics that confronted him in his practice: an acute epidemiological crisis; changing patient demands; a personal desire and practical need to synthesize diverse strands of tradition into a workable practice; and new forms of engagement with the body among Jiangnan physicians that sought to move away from long-standing cosmological frameworks of resonance towards an empirically grounded topography of bodily space. The historical confluence of these problematics inspired Ye Tianshi to imagine the body as a system of interlinking networks composed of larger conduits and successively smaller network vessels, and to define obstruction to free flow in these networks to be the major cause of disease.

To further undercut the modernist binaries that structure our understanding of Chinese medicine, I will also show that the body Ye Tianshi worked with is readily intelligible to modern readers, even if they know nothing of Chinese medicine; and yet, because this body is rooted in *tong* and the networks rather than biomedical anatomy or biochemistry, that it is also radically different. Or, to put it another way, precisely because the bodies of the patients that Ye Tianshi treated in his seventeenth-century Suzhou clinic were not materially all that different from those we inhabit today, their very materiality constrained his scope for innovation. But because he approached this materiality from a very different perspective to that of contemporary biomedicine or biochemistry, it offered him a vastly different space for innovation and intervention.

Given the specialist nature of the sources I work with, I must ask my readers to follow me patiently through a terrain of unfamiliar concepts, body parts, and practices in an exploration that will lead out, at the other end, onto new vistas on both the history of Chinese medicine and the comparative history of science and medicine. To help readers navigate this unfamiliar terrain I begin by first explaining the concept of *tong* and how it was used as a conceptual tool by Chinese medicine physicians. Next, I will introduce Ye Tianshi, my main historical actor, in relation to his environment and time. I will then cover in some detail the transformation of medical thinking and practice that occurred in the lower Yangzi valley between the late sixteenth and mid-eighteenth centuries, including a review of the concept of bodily “networks,” before examining Ye Tianshi’s own innovations. In the concluding section I will return to the issues raised in this introduction to ask what contributions my case study can make to the histories of medicine, science, and the early modern.

## The concept of *tong* 通 in Chinese medicine

Despite the outpouring of studies on the history of Chinese medicine over the past two generations, little attention has been paid to the concept of *tong*. The term is not listed, for instance, in the index to Hinrichs and Barnes’ recent comprehensive historical survey of Chinese medicine and healing.^10^ It is equally absent from the index to Manfred Porkert’s *Theoretical Foundations of Chinese Medicine*, from Shigehisa Kuriyama’s comparative analysis of Greek and Chinese medicine, and from Lloyd and Sivin’s study of science and medicine in early China and Greece.^11^ It is referenced once only in Paul Unschuld’s *Medicine in China.*^12^ The one exception is Nathan Sivin’s *Traditional Medicine in Contemporary China*, where the term is indexed both singly and in various compound terms; but this is largely because a considerable part of the book is a translation of a Chinese text.^13^

This absence of *tong* in the English language literature on Chinese medicine contrasts starkly with Yanhua Zhang’s ethnographic account of Chinese medical practice in contemporary China. Drawing on observations in a Beijing Chinese medicine hospital during the 1990s, Zhang places *tong* not only at the heart of Chinese medical practice, but the phenomenology of bodily experience in Chinese culture at large. “Chinese people are oriented to the sense of a smoothly flowing process, which is characterised by such images as *tong* 通 (open, through, extending, connecting, continuing, and flowing), *huo* 活 (alive, active, and flexible), or *shun* 順 (unobstructed, smooth). These images are positively valued by Chinese in their body as well as their social world.”^14^

Zhang’s findings about the importance of *tong* also accord with historical writings. If we examine a range of exemplary Chinese medical texts from the Han to the late imperial era, we find that they frequently mention *tong* as a goal of therapeutic intervention. In fact, it occurs at least as frequently as, if not more often than, the two goals of “supplementation” (*bu* 補) and “draining” (designated with two different Chinese characters瀉/泄, both pronounced *xie*), which historians and practitioners have long assumed to have been at the forefront of Chinese physicians’ minds throughout the ages (see Table 1).

**Table 1.** Occurrence of *tong* 通 vis-a-vis supplementation (*bu* 補) and draining (*xie* 瀉/**泄**) in key texts of the Chinese medical tradition.

Text	Tong 通	Supplement 補 bu	Drain (1) 瀉 xie	Drain (2) 泄 xie
*Inner Canon Basic Questions* 內經素問, second century BCE	112	61	67	112
*Important Formulas Worth a Thousand* 千金要方, Sun Simiao 孫思邈, 650	467	375	240	233
*Book to Safeguard Life Arranged According to Pattern* 類證活人書, Zhu Gong 朱肱, 1108	72	15	55	29
*Annotation and Explanation of the Discussion of Cold Damage* 注解傷寒論, Cheng Wuji 成無己, 1144	128	37	64	92
*Discussion of Illnesses, Patterns, and Formulas Related to the Unification of the Three Etiologies* 三因極一病證方論, Chen Yan 陳言, 1174	148	94	86	133
*Collection of Writings on the Mechanisms of Disease, Suitability of Qi, and the Safeguarding of Life as Discussed in the Basic Questions* 素問病機宜保名集, Liu Wansu 劉完素, 1186	128	55	98	125
*Treatise on Spleen and Stomach* 脾胃論, Li Gao 李告, thirteenth century	72	80	123	70
*Essential Teachings of [Zhu] Dan-Xi* 丹溪心法, Zhu Danxi 朱丹溪, 1481	146	251	153	176
*Systematic Differentiation of the Discussion of Cold Damage* 傷寒論條辨, Fang Youzhi 方有執, 1592	186	46	52	71
*Collected Works of [Zhang] Jing-Yue* 景岳全書, Zhang Jiebin 張介賓, 1624	117	338	136	875
Records of Pattern Discrimination 辯證錄, Chen Shiduo 陳士鐸, 1687	451	1,878	641	321
Precepts for Physicians 醫門法律, Yu Chang 喻昌, 1658	297	310	158	103
Comprehensive Medicine According to Master Zhang 張氏醫通, Zhang Lu 張路, 1695	1,367	2,098	1,145	527
Collected Writings on Renewal of the Discussion of Cold Damage 傷寒來蘇集, Ke Qin 柯琴, seventeenth century	94	49	81	17
Awakening of the Mind in Medical Studies 醫學心悟, Cheng Guopeng 程國彭, 1732	143	335	76	0
Case Records as a Guide to Clinical Patterns 臨證指南醫案, Ye Tianshi 葉天士, 1746	932	593	456	611
*Refined Medicine Remembered* 醫醇剩義, Fei Boxiong 費伯雄, 1864	115	92	100	20
Random Notes while Reading about Medicine 讀醫隨筆, Zhou Xuehai 周學海, 1898	151	254	128	156

If a simple word count represents a very crude measure for getting at the argument of a text, Table 1 suggests that just how much a given author valued *tong* relative to other treatment strategies varied considerably. That is, although *tong* does appear to express an important but widely neglected orientation within many different currents of Chinese medicine, it equally does not constitute its unchanging focus as Zhang appears to claim. Instead, we need to investigate how a concept like *tong* comes to be articulated within distinctive historical conjunctures and what problematics it resolves. This, in turn, demands of us to engage with the substantive worlds and bodies at stake in the thoughtfully assembled and deployed medical imaginaries of historical actors.

Historians of the subject broadly agree that a foundational moment in the emergence of Chinese medicine occurred when healers started to imagine the body/person and its relationship to the wider cosmos as being constituted by flow and movement. The term *tong* had already entered medical discourse during these formative stages of development in the Spring and Autumn era (ca. 771 to 476 BCE). In the phrase *tong shenming* 通神明, for example, *tong* meant “getting through to” or “passing into (another state),” namely that of “clarity of spirit” (*shen ming*). This was achieved through practices of self-cultivation that involved acquiring the ability to connect together the acupuncture conduits or *mai* 脈. It marks a phase in the development of medicine from one where such clarity was associated with external spirits entering the body and one where the personal spirit became individuated.^15^

In the two constituent works of the *Inner Canon of the Yellow Lord* (*Huangdi neijing* 黃帝內經), based on writings from the first and second century BCE, and widely considered since to be the foundational text of the Chinese medical tradition, *tong* conveys a range of interrelated meanings clearly derived from the above.^16^ In the senses of “openness,” “affording passage,” “penetration,” “passing through,” “communication,” and so on, it is used to describe the functioning of the conduits and networks (*jingmai* 經脈, *jingluo* 經絡), the unhindered flow of *qi* and blood through these, and the vitality that is thereby bestowed onto a person. The earlier self-cultivation practices that centered on *tong* as communication with the spirits were also integrated into emergent naturalist conceptions of the body. “Clarity of spirit” (*shenming* 神明) now depended on the capacity of the Heart visceral system and sense organs to afford unobstructed passage between the exterior and interior worlds and thereby allow the appropriate expression of emotions, including their ability to be manifested in a smooth and unhindered manner.^17^

The authors of the *Inner Canon* considered acupuncture, specifically the use of the fine (filiform) needle, to be the prime technology for “opening” (*tong* 通) and regulating the blood and *qi*. By the middle of the sixth century, “unblocking” (*tong* 通) had also become an integral strategy in pharmacotherapy, and “unblocking” formulas now constituted one of the ten types of prescription (*shi ji*十劑) listed in *Herb Pairs of the Lightning Lord (Leigong yaodui* 雷公藥對).^18^

Indeed, a wide range of medical writers now considered the unhindered flow of *qi*, blood, essences, and body fluids within the body, as well as unobstructed communication between interior microcosms and the macrocosm, to be synonymous with health and wellbeing. The first chapter of *Essentials of the Golden Casket* (*Jingui yaolüe* 金櫃要略) by the late Han author Zhang Zhongjing 張仲景 (150–219) forcefully expresses this view in its opening passages:

As all human beings receive the [same] five constants, they grow and develop [individually] because of the [universal] wind[like] qi. Though wind[like] qi can generate the ten thousand things, it also can harm them. This is just like water, which can float a boat but also overturn it. If the five viscera communicate freely (*tongchang* 通暢) with the original true [qi], a person is safe and calm. When they suffer visitations of evil winds, many of the afflicted die. Although there are all sorts of ailments they do not go beyond three types. The first is when evils contracted into the conduits and networks enter the viscera and bowels. These accord with internal locations. The second is when the blood vessels that connect the four extremities and nine orifices are clogged up and do not afford free passage (*butong* 不通). This happens when the skin is the location to be hit. The third is damage due to sexual indulgence, injury with metal knives, or wild animals.^19^

Beginning in the eleventh century, scholars and physicians gradually elevated Zhang Zhongjing to the status of the father of pharmacotherapy, making his works an obligatory reference point for later physicians, including Ye Tianshi.^20^ Modern Chinese medical dictionaries, therefore, still widely accept the two interrelated definitions of *tong* presented in this quote: first, read in a transitive manner, as the “openness” and, by extension, “unblocking” of the conduits and networks; second, read intransitively, unhindered physiological process and activity of any kind. They also, however, list a range of more specific meanings. When used to describe treatment methods, *tong* includes strategies such as “diffusing obstructions” (*xuan bi* 宣痹), “moving areas of stagnation” (*xing zhi* 行滯), “eliminating stasis” (*qu yu* 去瘀), and “purging” (*gongxia* 攻下) via the intestines or urination. “Unblocking the menses” (*tong jing* 通經), “unblocking the *yang*” (*tong yang* 通陽), “unblocking the vessels” (*tong mai* 通脈), and “unblocking the intestines and urination” (*tong bian* 通便) are just a few of the many things that physicians do when they *tong* in clinical practice.^21^ I suggest that because the general concept of *tong* could be applied to many different aspects of bodily function, it became a useful conceptual framework that doctors could apply to a diverse range of illnesses. This explains why so many doctors (as seen in Table 1) refer to *tong*.

**Figure 1.**
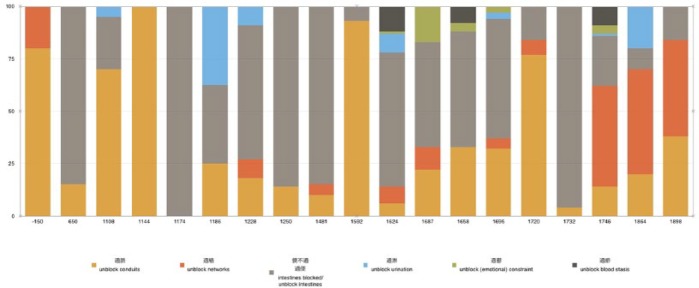
Change over time of the relative percentage of usage of *tong* 通 as a treatment method in the key texts compared in Table 2.

This hypothesis is confirmed by examining more closely the specific contexts in which different texts and authors use the term *tong*, as summarized in Table 2 and Figure 1.

**Table 2.** Occurrence of *tong* 通 as a treatment method in relation to specific objectives in key texts of the Chinese medical tradition.

Text	通脈 unblock conduits	通絡 unblock networks	便不通 通便 intestines blocked/ unblock intestines	通淋 unblock urination	通鬱 unblock (emotional) constraint	通瘀 unblock blood stasis
*Inner Canon Basic Questions* 內經素問, second century BCE	6	2	0	0	0	0
*Important Formulas Worth a Thousand* 千金要方, Sun Simiao 孫思邈, 650	7	0	39	0	0	0
*Book to Safeguard Life Arranged According to Pattern* 類證活人書, Zhu Gong 朱肱, 1108	14	0	5	1	0	0
*Annotation and Explanation of the Discussion of Cold Damage* 注解傷寒論, Cheng Wuji 成無己, 1144	12	0	0	0	0	0
*Discussion of Illnesses, Patterns, and Formulas Related to the Unification of the Three Etiologies* 三因極一病證方論, Chen Yan 陳言, 1174	0	0	11	0	0	0
*Collection of Writings on the Mechanisms of Disease, Suitability of Qi, and the Safeguarding of Life as Discussed in the Basic Questions* 素問病機宜保名集, Liu Wansu 劉完素, 1186	2	0	3	3	0	0
*Treatise on Spleen and Stomach* 脾胃論, Li Gao 李告, thirteenth century	1	0	6	0	0	0
*Confucians’ Duties to Their Parents* 儒們事親, Zhang Zihe 張子和, 1228	2	1	7	1	0	0
*Essential Teachings of [Zhu] Dan-Xi*丹溪心法, Zhu Danxi 朱丹溪, 1481	2	1	17	0	0	0
*Systematic Differentiation of the Discussion of Cold Damage* 傷寒論條辨, Fang Youzhi 方有執, 1592	14	0	1	0	0	0
*Collected Works of [Zhang] Jing-Yue* 景岳全書, Zhang Jiebin 張介賓, 1624	7	10	78	11	1	15
*Records of Pattern Discrimination* 辯證錄, Chen Shiduo 陳士鐸, 1687	4	2	9	0	3	0
*Precepts for Physicians* 醫門法律, Yu Chang 喻昌, 1658	8	0	13	0	1	2
*Comprehensive Medicine According to Master Zhang* 張氏醫通, Zhang Lu 張路, 1695	66	11	119	6	6	0
*Collected Writings on Renewal of the Discussion of Cold Damage* 傷寒來蘇集, Ke Qin 柯琴, seventeenth century	10	1	2	0	0	0
*Awakening of the Mind in Medical Studies* 醫學心悟, Cheng Guopeng 程國彭, 1732	1	0	25	0	0	0
*Case Records as a Guide to Clinical Patterns* 臨證指南醫案, Ye Tianshi 葉天士, 1746	13	45	23	1	4	8
*Refined Medicine Remembered* 醫醇剩義, Fei Boxiong 費伯雄, 1864	2	5	1	2	0	0
*Random Notes while Reading about Medicine* 讀醫隨筆, Zhou Xuehai 周學海, 1898	5	6	2	0	0	0

Table 2 and Figure 1 (which displays the information contained in Table 2 in a graphic format that allows for easier comparison) show that most authors employ *tong* when referring to pathologies and treatment of the *jing* 經, a term that can refer to acupuncture conduits but also to topographically defined bodily domains.^22^ The second most important context to which authors apply the term is describing and treating elimination via the intestines and urination (*tong bian* 通便).^23^ This changes radically with Ye Tianshi, the first author to give predominance to unblocking the networks. After that, authors begin to focus more attention on unblocking the networks, too, demonstrating Ye Tianshi’s influence. This raises the question as to why this shift from unblocking conduits or intestines and urination to unblocking networks occurred and what it implied. To this end, it is time to introduce the main historical actor of this paper and to situate him in the context of his time and his place.

## Ye Tianshi: a seventeenth-century Suzhou physician

Ye Tianshi was a third-generation physician whose family had moved from neighboring Anhui Province to Suzhou. Located in present-day Jiangsu Province, about an hour’s train ride from Shanghai, Suzhou was then one of the foremost economic and cultural centers of China. A modernizer and accomplished clinician whose style of prescribing would shape clinical practice in Jiangnan and beyond for generations to come, Ye Tianshi became a legendary physician even during his lifetime.^24^ He is particularly well known for creating new methods for diagnosing and treating febrile disorders at a time when epidemics were a major threat to public health. Ye also proposed new ways for treating miscellaneous disorders (*zabing* 雜病) like pain, debility, and emotional constraint, gynecological diseases, and pediatric disorders, thereby extending and refining previous understandings of bodily function in Chinese medicine writ large.

As a practicing physician who did not himself write any books, Ye Tianshi largely owed his legacy and influence to a series of texts compiled by students, admirers, scholars, and critics between the late eighteenth and mid-nineteenth centuries.^25^ Xu Dachun 徐大椿 (1693–1771), another influential scholar physician from the Suzhou region, published a critical edition of Ye Tianshi’s case records that, despite his many criticisms, contributed much to establishing Ye’s reputation.^26^

The way that different readers imagined Ye Tianshi over time was marked by two contradictory impulses. One depicts him as a genius physician who studied with at least seventeen different masters, who was exceptionally well read, able to transcend the one-sidedness of existing doctrines, and willing to go beyond established modes of medical practice wherever necessary. Simultaneously, however, his genius – unlike that of the great ancestor of pharmacotherapy Zhang Zhongjing – is viewed as ultimately limited to a specific geographical region and a specific set of medical problems. Hence, Ye Tianshi is primarily remembered as a key figure in the emergence of the “warmth disorder current” (*wenbing xuepai* 溫病學派), a new way of thinking about febrile and epidemic diseases that emerged in Jiangnan between the sixteenth and eighteenth century and that was widely viewed as closely tied to “Southern” climates and constitutions. Similarly, he is recognized as a leading representative of a distinctive Suzhou style (*Su pa*i 蘇派) of medical practice characterized by a preference for mild acting medicinals and low dosages.^27^ Few commentators view Ye Tianshi as someone who sought to push Chinese medicine in its totality in a new direction.^28^

This imaginary and its inherent biases, tensions, and contradictions were drawn into the debates that shaped the development of Chinese medicine over subsequent centuries and also left a significant imprint on its historiography.^29,30^ Against this background, an analysis of *tong* and the body’s networks as central problematics of Ye Tianshi’s medicine offers us the opportunity to take a fresh look at his innovations, unencumbered by the sediments of historical memory. This requires first, however, to situate his efforts even more clearly within the context of their place and time.

## Suzhou and Suzhou medicine during China’s long seventeenth century

Rather than looking at Ye Tianshi as an individual genius, he is best viewed as a product of his time, his medical perspectives resonating with the interlinking transformations of society, economy, culture, and medicine that originated in the eleventh century and reached their peak between the late sixteenth and early eighteenth centuries. These transformations were centered on the Jiangnan macroregion, with Suzhou a particularly important center. They included increased urbanization and literacy; specialization in agriculture, manufacturing, and trade; the gradual fusion of literati and merchant classes into a new expanding elite; the emergence of a cash-based market economy; and the development of a consumer society.^31^

As I have detailed elsewhere, one effect of these transformations was to reshape male gender identities amongst the Jiangnan elite.^32^ Norms of masculinity now included an emphasis on artistic sensibility, physical fragility, sexual passion, and even self-indulgence, over physical strength and prowess without, however, surrendering male social dominance over women.^33^ At the same time, successive Ming emperors also supported the cult of the war god Zhenwu 真武 and many elite males during the fifteenth and sixteenth centuries practiced martial arts, honed their military skills, and became actively engaged in military expeditions. In the domain of medicine, this was reflected in a renewed tendency to view medicine as akin to warfare, whose success depended on training in strategic agency rather than physical force.^34^ This, therefore, did not dislodge the widespread self-perception of Southerners as being physically fragile. Indeed, the same period witnessed a “cult of emotion” sweeping through elite society. This expressed itself in a booming market for emotionally charged literary works, frequently written by women, but also cherished by male readers whose status ambitions all too often did not match their actual achievements. The cult of emotions presented these men with new opportunities to reassert their elite status through displays of cultural sophistication.^35^

In the emerging consumer society of early modern Jiangnan, the male pursuit of cultural status also expressed itself in the collecting of books and works of art, and a newly emergent emphasis on connoisseurship of the ancient and authentic. The need to develop the critical attitude and skills that allowed one to distinguish the genuine from the fake gave rise to a more widespread reorientation away from the speculative metaphysics and search for universal moral principles that had characterized previous generations of elite thinkers, and towards a more empirically grounded understanding of and engagement with the workings of things in the here and now.^36^

In the medical domain, these transformations were reflected in a series of interlinking developments that shaped the context in which Ye Tianshi fashioned his own innovations. Alongside the expansion of book printing and collecting, which increased access to a wide variety of medical works from the past and present, there is also evidence for an intensification of direct interactions between physicians across families and lineages. Such trends facilitated the emergence of regional styles of practice. Many of the physicians that most immediately influenced Ye Tianshi’s thinking – Fang Youzhi 方有執 (1523–93), Wu Youke 吳又可 (1582–1652), Miao Xiyong 繆希雍 (1546–1627), Yu Chang 喻昌 (1583–1664), and Ke Qin 柯琴 (1662–1735) – were active in the immediate vicinity of Suzhou. Being able to study with seventeen different masters, likewise, is evidence not just of Ye Tianshi’s own open mindedness but also of a social environment that enabled a broadening of scholarship and the widespread exchange of ideas.

Building on – but also criticizing – each other’s work, and influenced by the intellectual currents of evidential research (*kaozheng* 考證) and the restoration of ancient learning (*fugu* 復古) that were then emerging across Jiangnan, these physicians embarked on a fundamental reevaluation of their medical tradition.^37^ They argued for replacing a scholastic emphasis on book learning with a more empirically oriented attention to things themselves. They employed new philological methods to radically reinterpret and reorganize the classical canons. They looked at the body in new ways, revisited *materia medica*, and detected important lacunae in existing medical knowledge.^38^

Consumers of medical services, meanwhile, demanded attention to real or imagined differences in bodily constitutions and the healthcare problems of their day. These included a series of severe epidemics that occurred during the late sixteenth and early seventeenth centuries and intensified what Marta Hanson has termed an “epidemiological crisis” – an increasing uncertainty amongst physicians as to whether existing doctrines regarding the causes and treatment of such disorders were sufficient.^39^ At the other end of this spectrum was the new nosological category of “emotion-related disorders” (情志病 *qíngzhì bing*) that emerged in the medical writings of the time.^40^ In the minds of mainstream physicians, both of these problems were associated with disturbances of flow.

Doctors had long viewed epidemic febrile disorders as caused by “evil *qi*” (*xieqi* 邪氣) penetrating into the body and then obstructing the movement and circulation of the body’s own “upright *qi*” (*zhengqi* 正氣), blood (*xue* 血), and body fluids (*jinye* 津液). What was increasingly up for debate, however, was the nature of this evil *qi* and its location in the body. Cold and wind had traditionally been viewed as the most important pathogenic forces, which could be transformed into heat once they had entered the body. Furthermore, by the thirteenth century a consensus had emerged that this evil *qi* lodged in the acupuncture conduits and their associated organ systems as mapped in the *Inner Canon*, and it was from these structures that it needed to be expelled. By contrast, the physicians that influenced Ye Tianshi had begun to focus on the possibility that heat could enter the body directly; or, even more revolutionary, that there were potentially other “miscellaneous *qi*” (*zaqi* 雜氣) that acted as specific causes for a wide range of different diseases.^41^ Simultaneously, they proposed new ways of understanding the topographical organization of the bodily space that evil *qi* could invade and afflict.

To this end, these physicians emphasized the difference and separation between the physical structures of the “bodily shell” (*quqiao* or *quke* 軀殼) – composed of skin, bones, conduits, vessels, muscles, and sinews – and the body’s internal organs.^42^ While earlier models of epidemic disease had relied on notions of resonance (*ying* 應) and systems of correspondence, Ye and likeminded Jiangnan doctors now emphasized explanations of disease causation and therapeutic action that paid attention to the physical movement of things through bodily space and to the ways in which local factors influenced this movement. These changes were part of larger medical trends, as evidenced in new understandings of emotion-related disorders, which shifted away from reading the cosmological patterns that structured the universe at large (and, ideally, guided moral behavior into the body), towards notions of contiguous causation ^43^ The notion of “constraint” (*yu* 鬱) is exemplary here. It originally denoted a hemming in of the physiological flow of *qi*, blood, and body fluids, and doctors recognized multiple possible manifestations and causes. From the mid-fifteenth century onward, constraint became increasingly attributed to emotional causes and the fragile constitution of the southern gentry, as summarized here by the court physician Xu Chunfu 徐春甫 (1520–96):

Constraint is a disorder of the seven emotions. Therefore, eight or nine out of ten patients suffer from it. … Chronic constraint manifests in innumerable types of disease. Men who have it become deficient and cowardly or manifest with dysphagia and constipation, bloating or abdominal distension. Women who have it stop having their periods, or manifest with miscarriage, uterine bleeding, or general debility. Treatment strategies must be able to interiorly nourish, before unblocking constraint and regulating according to the presentation.^44^

Historians of medicine have not yet examined the specific reasons that underlie these shifts. However, they are consistent with an increasingly critical attitude among intellectuals of the time regarding cosmology and the correlative thinking that undergirded it.^45^ There also appears to be some overlap with the developments that Kuriyama (1997) has documented for Edo period (1615–1868) Japan. According to Kuriyama, the rapid commercialization of Japanese society during this time, which depended on the circulation of money and goods, was mirrored in increasing concerns about problems of stagnation and blockage within the human body. Interestingly, these Japanese concerns match the emergence of similar anxieties during the industrial revolution in the West.^46^ Furthermore, Japanese physicians of this period blamed problems of *qi* constraint on idleness, affluence, and declining opportunities for venting emotional frustration through more outright physical aggression.^47^

## *Tong* as key to Ye Tianshi’s style of medical practice

In the previous section, we saw that Ye Tianshi was a key figure in attempts to rethink the treatment of epidemic disorders and fevers in seventeenth-century China. He made equally influential contributions to the discourse on constraint, describing, for instance, how emotions produce physical symptoms that can be read on the body’s exterior:

“Stagnation, whether present in the body or the organ systems must have visible manifestations of tension. *Qi* by its nature has no form but in the course of constraint the *qi* gathers together. This gathering together makes it appear to possess a form even if in reality it has no material substance.”^48^

This passage provides an example of Ye’s concern with flow and movement (of *qi*, blood, body fluids, as well as pathogens), and his view that it was the key to understanding and intervening in bodily processes. A close reading of the case histories collected in his *Case Records* also shows that this concern with flow – articulated in terms of the concept of *tong* – underpins Ye’s approach to a wide range of clinical problems. In this section I will outline Ye’s innovations and how they built on and deviated from existing practices. Taking Ye Tianshi’s practices seriously requires delving into its technical aspects in some detail.

The first issue is to understand how Ye envisioned the concept of *tong*. The term *tong* occurs a total of 935 times throughout Ye’s *Case Records*,^49^ conveying four distinct but interrelated meanings. These are:

i. To refer to body parts, functions, organs, or substances that suffer from an “obstruction of free flow” (*bu tong* 不通) that can then be rectified by a strategy of “facilitating flow” (*tong* 通). The list of locations, functions, and things to which this strategy can be applied is extremely long. In Ye’s *Case Records*, it includes stools, urination, semen, menstruation, *qi* and blood, *yang*, the muscles, the diaphragm, the different internal organs, rotten matter and pathogens, things that have no form, the *qi* dynamic of the Triple Burner, the nose, the emotions, spirit, the sense organs, and, of course, the conduits and networks.
ii. To describe a distinctive treatment method. As such, *tong* is distinguished from methods or processes that do not facilitate flow, such as firming, holding, congealing, tonifying, and nourishing. At the same time, *tong* is also differentiated from more forceful methods of unblocking such as purging, which implies the use of harsh medicinals. This second meaning of *tong* can be viewed as a subclass of the first, and more general, meaning.
iii. To describe an attribute of certain medicinals or the action of specific formulas. For instance, some medicinals are *tong* (unblocking) while others are cloying, and different formulas can also be used to *tong* (unblock) different bodily spaces or structures.
iv. To describe states and phenomena that lie outside strictly medical concerns, such as “flexibility and adaptability” (*bian tong* 變通), or “connection” (*guan tong* 貫通).

Ye Tianshi thus uses *tong* in both a transitive and intransitive manner. It denotes a state of affairs (something flows or is blocked), but also the capacity of a thing or action to block or unblock specific locations and physiological flows. However, because Ye’s *Case Records* contains few passages that explicitly discuss his conceptualization of *tong*, these meanings must be extracted patiently from scattered comments and the actual use of formulas and medicinals. One of the most theoretically explicit passages is a commentary by Hua Yutang 華玉堂, one of the book’s editors, who explains that free flow is central to the treatment of pain:

The ancients widely used the character “*tong*” as one of the most beneficial treatment methods. The character “*tong*” must not be understood incorrectly as attacking-purging or promoting urination. Instead, it refers to providing free passage to qi and blood so that there is no pain. However, one must differentiate its specific location in the *qi* or blood aspect. At the *qi* aspect one moves the qi. One must not employ strong medicinals for a light disorder. When it is in the blood aspect, in attacking and moving the blood one must treat it together with the *qi*. This is what is meant by when the *qi* moves the blood follows.^50^

While this is Hua’s interpretation, rather than Ye Tianshi himself staking a theoretical claim, attentive reading of this passage nevertheless allows us to tease out four core attributes of *tong* as a treatment strategy.

First, Ye’s therapeutics draw a clear distinction between *tong* as “providing free passage to *qi* and blood” throughout the body and the main way that the term was used in earlier medical discourse, namely to unblock the intestines and urination (see Table 2). Ye does not abandon this earlier usage, which he still employs in specific contexts, but he emphasizes the new, broader meaning of *tong* to open new possibilities for therapeutic intervention.

Second, the concept of free flow embodied in this new sense of the term *tong* is linked explicitly to the use of milder medicinals and formulas. That is, Ye’s signature “light and nimble” Suzhou style directly embodies *tong* as a core clinical strategy while also responding to distinctive consumer demands.^51^

Third, while the consequences of a lack of free flow can manifest throughout the entire body, *butong* 不通 describes a single core pathology: the inability of *qi* (or *yang*) functions to flow freely and thus enable movement and transformation of blood (or *yin*) stuff. Therapeutically, this translates into an emphasis on ensuring unobstructed function (*tong*) rather than supplementing (*bu*) the material stuff that underpins such function. These are the principles that guide Ye’s recommendations for treating abdominal distension, for example: “Regarding the use of warming supplementation in cases of deficiency cold distension disorders, a review of our forefathers’ prescriptions invariably shows them to be composed of unblocking (*tong*) strategies. For if *yang* flows freely turbid *yin* does not congeal. Abiding only by supplementation risks dulling [the functions of] the middle burner.”^52^

Fourth, to successfully treat pathologies of free flow, one should employ new diagnostic strategies based on an improved understanding of *yin-yang* logic and its application to the components of the body and their interactions. As Ye states, “The character *tong* must be studied in relation to *qi* and blood, *yin* and *yang*, for this is the key to diagnosis.”^53^ Without going into excessive clinical detail, this statement implied several interrelated shifts in diagnostic practice, of which the following are the most important.

First, at the most general level, Ye adds a heightened concern for *tong* and the movement of stuff to existing medical practice. Expressed in the language of yin-yang logic, this leads Ye to categorize disorders into two types: those that pertain only to the failure of that which moves (*yang*), and those that involve pathologies of that which is being moved (*yin*). The former group comprises so-called “*qi* sector” disorders, while the latter comprises those that are involved or are located within the blood (*xue* 血) sector. The terms *qi* and blood are both used in a narrow and a wider sense, where *qi* comprises various modes of movement (mechanical, thermal, diffusional) embodied in distinctive types of *qi* – like protective *qi* (*weiqi* 衛氣), gathering *qi* (*zongqi* 宗氣), or *yang qi* 陽氣 – and blood can stand for all kinds of stuff – like blood or body fluids (*jinye* 津液). Ye employs *qi* and blood (sector) differentiation in internal medicine but also in the treatment of febrile disorders. There it becomes the foundation of Ye’s four sector (*wei qi ying xue* 衛氣營血) schema for differentiating warm pathogen disorders (*wenbing*), namely the innovation that Ye is most famous for today.^54^

Second, in the domain of internal medicine, Ye applies his new focus on *tong* to the pathologies of the organ systems, drawing a clear distinction between his approach and traditional approaches that relied on correlative five-phase (*wu xing* 五行) thinking. “The nature of the viscera is [to suffer from] thieving or over-control disorders [mediated by five-phase relationships]. This does not apply to bowel disorders whose function is based on free flow (*tong*). The character “*tong*” does not, however, refer to attacking purgation.”^55^

This passage is loaded with challenges to existing orthodoxy. Emphasizing Ye’s new reading of *tong* in relationship to both physiological bowel function and its opposition to purging treatments, it also inverts long-established hierarchies between viscera (*zang* 臟) and bowels (*fu* 腑). The term “bowels” here refers to the hollow organs of the body – such as the Stomach, the Intestines, or the Gallbladder – as opposed to the solid “viscera.” In the five-phases-based correlative thinking that underpins the *Inner Canon’s* cosmologically integrated body, the hollow bowels had hitherto been subsumed into larger systems of functions dominated by the solid viscera. This is reflected, for instance, in Manfred Porkert’s translation of *zang* 臟 as “visceral systems of function.”^56^ The Liver as representative of the wood phase within the human microcosm, for instance, included the Gallbladder as its associated bowel, but also other body parts like the nails, eyes, sinews, and tears.

While pathologies of the bowels were widely discussed in the literature, they were rarely accorded the same importance or status as pathologies of the viscera. One notable exception was the treatment of externally contracted febrile disorders. The then dominant cold damage (*shanghan* 傷寒) approach grounded in the work of the Han dynasty author Zhang Zhongjing (mentioned previously) had always paid attention to the removal of evil *qi* and its elimination via the stools, urine, or sweat and, therefore, also to obstruction of the bowels. Ye Tianshi fully embraced this approach, while also extending it to a range of newly conceived pathologies, specifically those caused by the direct penetration of warmth (*wen* 溫) and damp warmth (*shiwen* 濕溫) into the body through the nose and mouth.

## Conduits and networks

Having shown that *tong* was central to Ye Tianshi’s rethinking of medical practice across different domains, I now examine how the network vessels (*luomai* 絡脈) constituted a core bodily mediator in his conception of free flow and how they thus emerged as a main locus of pathology. Like *tong*, the concept of the networks dates back to the formative period of Chinese medicine, when structures known as *mai*, which were imagined to hold *qi* and blood, gradually became the foundation of new therapeutic practices.^57^ During this time, conceptual correlations between the flow of water in the earth and the flow of blood and *qi* in the body, coupled with an awareness of the existence of different types of blood vessels, led to the idea of a network (*li* 理) of vessels existing within the human body.^58^ By around the second century BC, when the constituent writings of the *Inner Canon* were compiled, the structure of this system had been worked out in considerable detail. A series of main conduits (*jingmai* 經脈) that traversed the body vertically were linked by secondary network vessels (*luoma*i 絡脈) extending predominantly in a horizontal direction.^59^ The network vessels, furthermore, included vessels of different sizes. Fifteen larger network vessels led off directly from the main conduits, while tertiary networks (*sunluo* 孫絡) branched off from these into the tissues of the skin and flesh.

Like the main conduits, the network vessels held and facilitated the flow of *qi* and blood. The *Inner Canon* describes how engorgement of tertiary network vessels by stagnant blood could become visible at the surface of the body and how this excess could be removed by blood-letting. In another passage, damage to the *yang* networks (*yangluo* 陽絡) is linked to nose bleeds, while that of the *yin* networks (*yinluo* 陰絡) manifests as blood in the stools. Compared to the main conduits, the network vessels were located more closely to the body’s external surfaces, and they were thus also imagined as important transmitters of evil *qi* that entered the body from outside. Pathogens first penetrated the skin, from where they passed into the tertiary networks. Once these were full and could no longer contain the evil *qi*, it would pass into the larger networks, then the main conduits, and finally into the viscera and bowels.^60^

Acupuncture, which was the dominant therapeutic method employed in the *Inner Canon*, could treat pathologies of the networks directly via bloodletting or indirectly by needling points assumed to be connected to these networks. Contemporary Chinese medical authors give us the impression that these ideas about the vessels were seamlessly integrated into pharmacotherapy. However, their depictions tend uncritically to project modern conceptualizations of network vessel pathologies (themselves strongly influenced by Ye Tianshi) back into earlier periods without accounting for the complex epistemological work that allowed these associations to be constructed in the first place. Ye Tianshi himself was more historically astute when he noted, “I searched the entire medical literature but it did not previously discuss network vessel disorders.”^61^ To him, this lack of attention to the networks constituted an important lacuna in medical knowledge that prevented doctors from effectively treating a wide range of everyday clinical problems, including pain and fevers. Thus, he lamented that, “[As for] physicians who do not understand the method of treating the networks, the longer they treat, the worse [the condition] gets.”^62^ The historical question that needs answering, therefore, is why Ye’s search for therapeutic solutions led him to focus on physiological structures that had been known for well over fifteen centuries, yet had not hitherto been a main concern for practicing physicians.

## Ye Tianshi on networks and network systems

To answer this question, one must understand that by the time Ye Tianshi began practicing medicine, Jiangnan physicians were already well advanced in their reconceptualizations of bodily space, and that these innovations themselves had begun to raise new conceptual and clinical problems. Specifically, I argue that Ye Tianshi’s thinking was profoundly influenced by the attention that physicians were now paying to the bodily shell, both as a zone of transition through which evil *qi* penetrated into the body, as well as a site of pathology in its own right. The connections that Ye envisioned between bodily shell, networks, and pathogens are evident in the passage below:

Internally caused [disorders] where damage is due to the seven emotions invariably first involve the viscera and bowels and then extend to the muscular body. Externally caused [disorders] due to contraction of the six [evil] qi invariably first involves the muscular body and only later enters the viscera and bowels. These are invariable principles. [For disorders] in the interior examine the Diagram of Internal Pathways (*Neijingtu* *內經圖*). For those in the exterior look at a map of the conduits and networks.^63^

The term “muscular body” (*jiqu* 肌軀) in the above quote from Ye’s case records corresponds to the concept of the “bodily shell” (*quke* 驅殻) that physicians like Fang Youzhi had recently introduced into medical discourse. In another passage, Ye notes that, “[i]n disorders of the bodily shell, the ancients always employed dispersing and moving prescriptions that promoted free flow.” Here, Ye finds justification for employing *tong* as the central treatment principle for pathologies of the bodily shell. Furthermore, this bodily shell – envisioned as extending from the skin on the exterior to the linings of the abdominal cavity that housed the body’s viscera and bowels – was also the region in which the conduits and networks were traditionally located. It was then only a small step for Ye to connect these two observations and to conclude that *tong* methods should target the networks.

Ye’s attention to the networks also echoed investigations by other doctors. Notably, the importance of these networks had been emphasized by Yu Chang, one of the most influential medical thinkers of the seventeenth century and a direct influence on Ye Tianshi. Yu argued that within the overall system of the *mai* or conduits, the networks formed a reticular network (*gangluo* 网络) composed of four distinct levels of organization: (i) there were twelve main network vessels (*daluo* 大絡) that branched off from the main conduits; (ii) these main network vessels then split into 180 connecting networks (*xiluo* 系絡); (iii) these connecting networks further ramified into 180 coiling networks (*chanluo* 纏絡); (iv) these coiling network further ramified into 34,000 tertiary networks (*sunluo* 孫絡). These networks intertwined together to form a system of “circulatory pathways” (*xunhuan daoluo* 循環道絡) that interconnected all the different organ systems, now imagined as one large network structure.^64^

Yu Chang also discussed pathologies of the conduits and networks in terms of stagnation and blockage, and he distinguished between two types of disease progression. In the first, external evils like wind and cold entered the body from the outside and progressively moved from the tertiary networks into the main conduits. In terms more readily understood by modern readers, what he meant was that cold first constricts flow in the smallest and most external networks, but it also may eventually affect flow in the larger conduits. The second type of progression was concerned with internally generated disorders, which Yu Chang defined as those caused by pathological changes of the stuff that flows in the conduits: static or polluted blood, constrained or knotted *qi*, phlegm fluids, or accumulations and concretions. Yu describes how this pathological stuff first fills up the larger conduits, but consequently also moves into successively smaller networks, that is, moving from the inside to the exterior. However, precisely because this pathological stuff has form, it cannot be expelled from these networks to the exterior, and it therefore ultimately leads to congestion of the entire network system. This congestion can only be relieved by treatment methods such as bloodletting that physically remove the pathological stuff. ^65^

**Figure 2.**
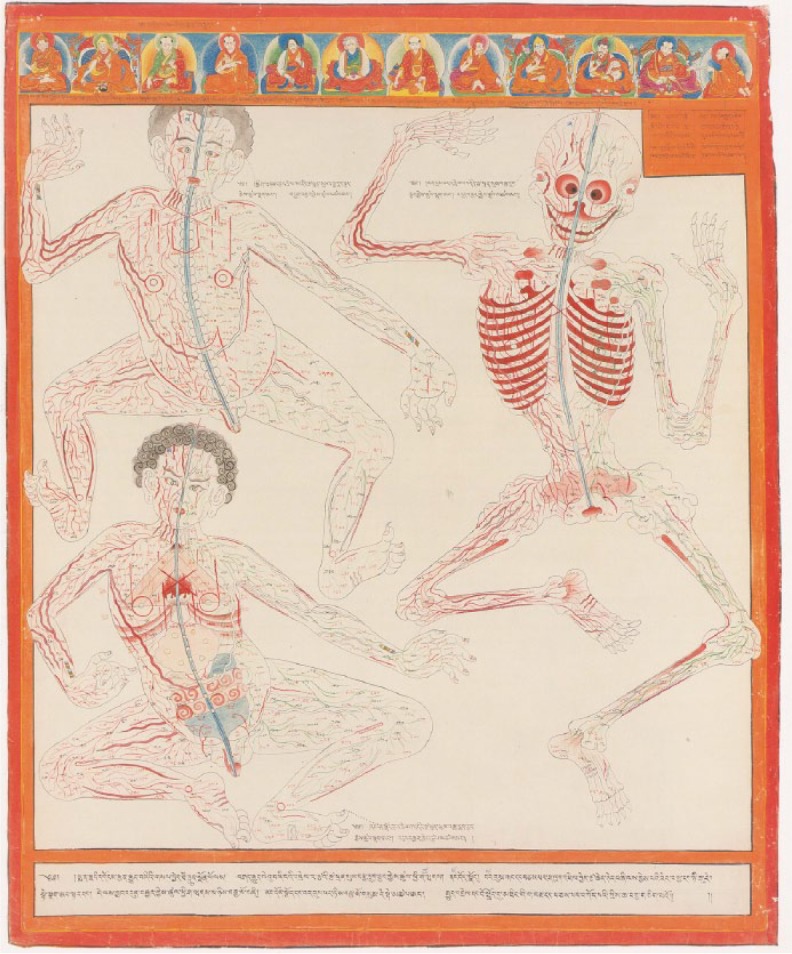
The body’s circulatory pathways as depicted in the Blue Beryl treatise of Sangye Gyamtso (1653–1705).^68^

Yu Chang himself moved within scholarly circles connected to the Jesuits, and at least one of his case records demonstrates that he assimilated Western anatomical knowledge into his innovative understandings of bodily function.^66^ He also was a practicing Buddhist, however, who had been a monk for part of his life and who devoted considerable energy to introducing Buddhist ideas into Chinese medical practice. He clearly was familiar with Buddhist medical texts from a variety of traditions.^67^ Unlike the Chinese medical literature, which only ever depicted the twelve main conduits, Buddhist representations of the body contain graphical representations of all the body’s circulatory pathways (Figure 2).

It is no longer possible to ascertain what specifically drew Yu Chang’s attention to the networks and their structure. His engagement with other medical traditions, however, stems from an intellectual open mindedness and curiosity typical of his time and of the circles within which he moved. His associates included leading seventeenth-century intellectuals like the poet and literary critic Qian Qianyi 錢謙益 (1582–1664) and the polymath Fang Yizhi 方以智 (1611–71), who combined a classical Confucian education with active and very practical interests in Buddhism, Daoism, Western learning, and also medicine.^69^

An interest in exploring ancient knowledge through a new focus on bodily structure, furthermore, was widespread and not limited to the Suzhou region. Zhang Zhicong 張志聰 (1616–74), an influential physician from neighboring Zhejiang, also drew attention to the role of the networks, specifically in the circulation of constructive (*ying*營) and protective (*wei* 衛) [*qi*], physiological entities widely considered to play a key role in the body’s ability to engage with invading evil *qi*. “The secondary networks communicate with the skin in the exterior and connect with the conduits in the interior. They are what mediates the free flow of constructive and protective. Hence, when an evil settles there, it hinders the constructive and protective.”^70^

Zhang Zhicong headed a discussion group on medical topics in Hangzhou and over fifty physicians considered themselves to be his disciples. He rejected some of Yu Chang’s innovations, and there is no evidence that he exerted any direct influence on Ye Tianshi. Zhang’s writings, nevertheless, attest to a more general interest among physicians at the time in discovering how physiological functions were related to concrete bodily structures. ^71^

What Ye Tianshi added to this emergent discourse, above all else, were new therapeutic practices. However, unlike Yu Chang and Zhang Zhicong, who wrote for an audience of fellow literati, Ye did not present his ideas in a systematic manner. His medical philosophy, therefore, needs to be carefully assembled on the basis of brief and generally unconnected remarks dispersed throughout his case records. What these records reveal, I argue, is that Ye perceived of the body in similar ways to Yu Chang, namely as constituted of an increasingly fine reticular network that fills bodily space and mediates the (ideally) free flow of *qi*, blood, and body fluids. Branching from the main conduits that communicate between the organs and the body’s external shell, network vessels extend in two directions: the *yang* networks (*yangluo* 陽絡) extend exteriorly towards the skin, while the *yin* networks (*yinluo* 陰絡) extend interiorly. Even the bodily organs appear to consist of viscera (*zangluo* 臟絡) and bowel networks (*fuluo* 腑絡).

Going beyond Yu Chang, Ye Tianshi thus appears to imagine the networks not merely as the interconnecting pathways of bodily circulation, but as physically constituting the body’s organs and tissues themselves. For example, he diagnoses a case of testicular swelling as due to turbid fluid collecting in the Liver networks (the testicles in Chinese medicine are traversed by the Liver conduit).^72^ Similarly, he explains rectal bleeding in a teenager as caused by blood leaking through gaps in the Intestinal networks.^73^ As for epidemic febrile disorders, Ye explains that the Lung network vessels connect to the nose and thus can be entered by warmth evils during inhalation, a description that evokes images of the mucosal structures that line the airways.^74^ From the Lung networks, invading evil *qi* can easily enter into the neighboring “Heart Enveloping Networks” (*xinbaoluo* 心包絡). Conventionally translated as Pericardium, the visible blood vessels that envelop not merely the heart but also the bronchi and bronchioles suggest the material reality that Ye might have had in mind. Transmission of evil qi from the Lungs to the Heart Enveloping Networks moreover constitutes a “sinking” (*chen*沉) from the *qi* aspect into the blood aspect. It indicates a worsening of pathology that is now imagined as movement from one discrete physical space to a contiguous one.^75^

In such explanations, Ye Tianshi essentially replaces existing explanations of disease processes built on logics of resonance and correlative thinking with explanations based on perceived pathologies of structures and spatial relationships. To give a concrete example, the *Inner Canon* famously described the Spleen as prone to damage from dampness. This is because, according to the five-phase system of correlative thinking, both Spleen and dampness resonate with the phases of the earth. Beyond these five-phase correlations, no further explanation is necessary to explain why dampness injures the Spleen. For Ye Tianshi, however, Spleen dampness consists of physical congestion of the Spleen networks by excess fluids. Lack of appetite and no interest in food are the symptoms that signal this physical repletion. Drinking warm soup will bring momentary relief to a patient because it adds *yang* warmth to the existing excess of *yin* fluids, thereby assisting the Spleen’s functions of movement and transformation. In the long term, however, warm soup exacerbates the problem. It not only increases fluids without fundamentally resolving the Spleen’s inability to assimilate and move them; it also adds heat to the existing constraint, turning dampness into damp heat, a problem that Chinese physicians consider much more difficult to resolve.^76^ In another, related reconfiguration, Ye abandons five-phase explanations for the presence of Stomach symptoms with concurrent Liver pathology. Instead, these are now explained by Liver *qi* entering the Stomach networks, where it does not belong, causing epigastric pain. The anatomical proximity between Liver and Stomach and the resultant physical intertwining of their networks is the key factor that facilitates this pathological encroachment.^77^

Indeed, in many of Ye Tianshi’s cases, it is precisely this intertwining of networks that constitutes the explanation for the progression and development of disease. Externally contracted evil *qi* initially stalls in the most superficial networks within the skin (the main entryway for cold and wind), in the airways (the main entryway for warmth), and in the upper digestive tract (the main entryway for damp heat toxins). As described by Zhang Zhicong above, these are the superficial bodily regions in which protective (*wei*) and constructive (*ying*) *qi* circulate to provide a first line of defense against invading pathogens. In fact, the characters *wei* 衛 and *ying* 營 and the imaginary they evoked were borrowed directly from military discourse, newly fashionable among seventeenth–century physicians. As Unschuld and Tessenow explain, “The military here includes troops that guard through patrolling and others that wait in camps to be mobilised for action. … The patrolling guards (*wei 衛*) were seen as fulfilling a *yang* function, the stationary, walled-in troops in a camp (*ying 營*) were seen as ideal to signify a *yin* function.”^78^

In Ye Tianshi’s approach, this military imaginary is structurally mapped onto the network vessels associated with the Lungs and the Heart Enveloping Network. From the most superficial network vessels in the skin and the airways (the *wei* sector), pathogens can either move into the larger conduits traversing the chest and the lungs (the *qi* sector), or they can be transmitted into the superficial networks of the Heart Enveloping Network. There they impede the movement and mobilization of the circulating blood and the Heart (the *ying* sector), and finally damage the physical structures of the body itself (the *xue* or blood sector). Another possibility is for evil *qi* located in the chest to move deeper into the diaphragmatic region and from there into the stomach and intestines. Yet another possibility is a progression from the superficial networks in the mucosa of the digestive tract into the circulating blood and the internal organs.

Each stage in the progress of an externally contracted disorder is thus explained in terms of specific networks and conduits filling up with evil *qi* until they can contain no more. In each stage, the free flow of *qi*, blood, and body fluids that would, under normal circumstances, prevent such congestion becomes blocked (*butong*不通). The appropriate treatment strategy, accordingly, is to prevent progression of the disorder by actively restoring free flow within given networks, to facilitate wholesome transit between networks, and to promote elimination of pathogenic stuff like static blood or phlegm towards the exterior through the stool or urine.^79^

Internally generated disorders involve the same networks but emerge through different modes of causation. One possibility is when an organ loses control over its metabolites. For example, fluids that should be managed by the Kidneys may pathologically accumulate in the Lung networks and conduits, and *qi* ordinarily managed by the Liver may trouble the networks of the Stomach. A second process involves pathologies of free flow that progress from constraint and stasis to visible or palpable accumulations. I already highlighted the importance of constraint for the understanding of emotional disorders in seventeenth-century China, but another widely recognized manifestation of flow pathology was pain. Ye Tianshi was the first physician to distinguish systematically between lack of free flow in the main conduits and the collateral networks in the treatment of pain. Even today, one of the most famous and often quoted passages from his *Case Records* states that, “Lingering pain invariably entails an entering [of the disorder] into the networks” (*jiu tong bi ru luo* 久痛必入絡).^80^

In this passage, the character *jiu* 久, which I have translated as “lingering”, implies that such pathologies are more difficult to treat. This is because the increasingly small size of the networks makes it more and more difficult to eliminate obstructions from them. The solution is to promote free flow with the help of medicinals that are only moderately *yang* in nature (because excessive warmth and acridity would dry up the fluids and blood contained within the networks and thus exacerbate existing stasis), and that are moist but not sticky (thus adding slipperiness without being cloying). For, “[o]nly by means of promoting free flow can lingering evil [*qi*] be uprooted,” and this, according to Ye Tianshi, is also what is meant by the saying that “when there is free flow there is no pain.”^81^

Ye’s *Case Records* thus show a great deal of overlap in the treatment of externally contracted and internally caused disorders. This is not surprising if both are imagined as located in the same bodily terrain, a terrain that is now imagined as composed of intertwining reticular networks. The difference is simply that externally contracted pathogens move from the most superficial networks into the larger conduits and organs, while internal damage disorders involve blockage in the larger conduits, if they are acute, and stasis in the network vessels, if they linger.

## Conclusion: Early modernity, problem spaces, and the materiality of the body

Although modern readers may consider the language and concepts of seventeenth-century Chinese medicine to be esoteric, the clinical logic of Ye Tianshi’s conceptualization of disease as rooted in obstructions of the body’s circulatory networks can easily be translated into contemporary biomedical imaginaries. Like the febrile disorders encompassed by Ye’s “warmth disorders” disease, acute infections do tend to progress from initial symptoms like fever, body pain, or nasal congestion (symptoms located in the bodily shell) to potentially more serious pathologies at the level of the internal organs. In internal medicine, acute pain either resolves spontaneously or turns out to be rooted in serious disorders, such as when a gall stone obstructs the bile duct or an embolus the circulatory system. Liver cirrhosis and the gradual narrowing of our arteries, on the other hand, tend to progress more slowly, and they stubbornly resist not only the efforts of Chinese medicine but also those of modern biomedical science. The reason, as Ye Tianshi noted in his own time, may be that “As for physicians who do not understand the method of treating the networks, the longer they treat the worse [the condition] gets.”^82^

**Figure 3.**
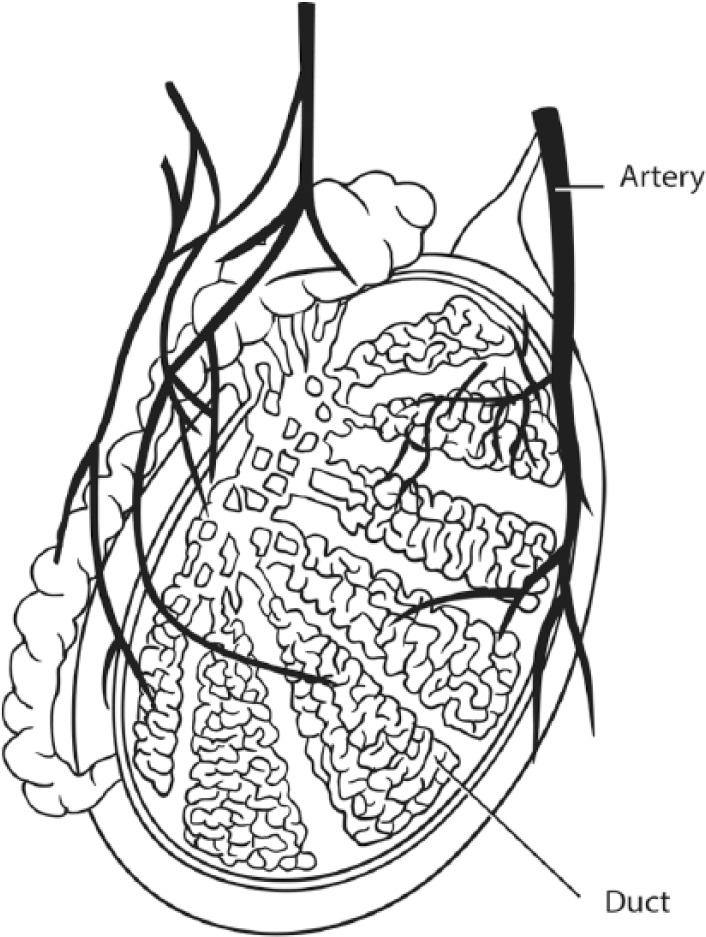
Diagram of a human testis.

**Figure 4.**
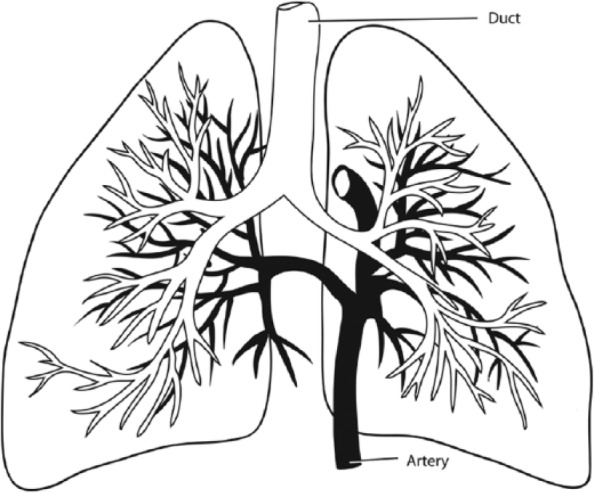
Diagram of the human lungs.

**Figure 5.**
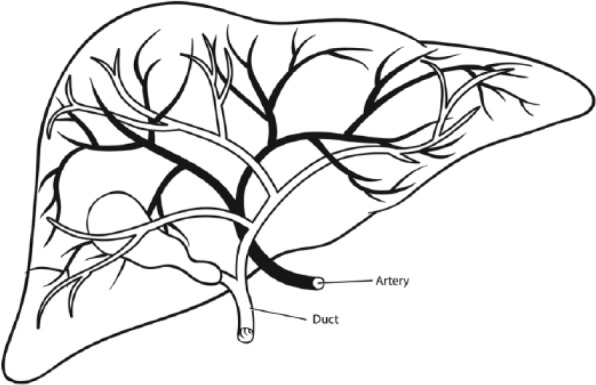
Diagram of the human liver.

**Figure 6.**
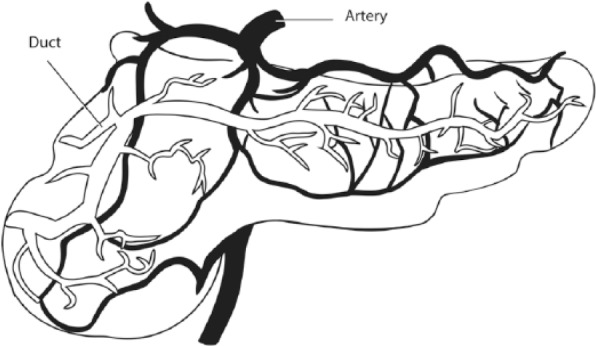
Diagram of the human pancreas.

**Figure 7.**
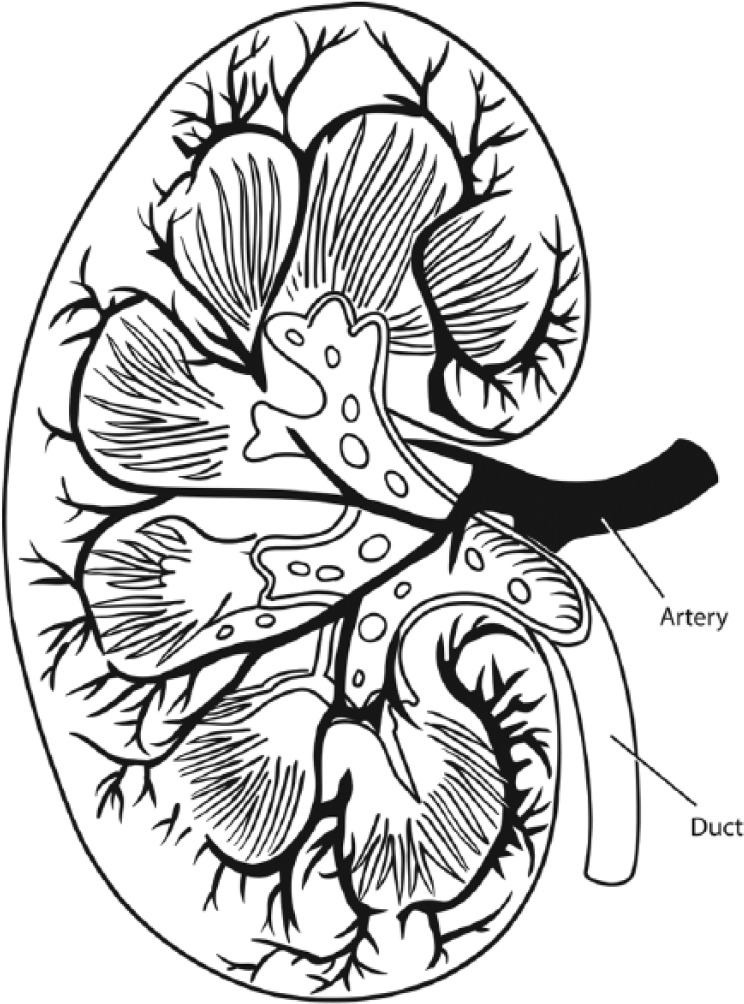
Diagram of the human kidneys.

Likewise, I think it is possible to translate Ye Tianshi’s understanding of bodily space into one more familiar to contemporary readers by looking briefly at the basic structure of our internal organs. The liver, kidneys, lungs, pancreas or, indeed, the testes that Ye Tianshi treated in the cases described in the previous section are all constituted by reticular networks, where one or more central tracts branch off into smaller and smaller secondary tracts. These tracts, furthermore, are enveloped by blood vessels, which themselves form a system comprising large central tracts and ever-finer networks linked to the heart. In between are areas of transition where the boundary between one anatomical structure and another begin to dissolve, a space known to Ye Tianshi and his contemporaries as being “half interior, half exterior” (*ban biao ban li*半表半里). If we likewise view the *wei* and *qi* sectors through which externally contracted evils progress at least in part as mapping onto bodily tracts that hold and carry air, bile, urine, or sweat, and the *ying* and *xue* sectors, into which these evils ultimately penetrate, as mapping onto the structures and content of the circulatory system, Ye Tianshi’s body and that of modern anatomy perhaps no longer seem worlds apart (Figures 3–7).

Ye Tianshi’s reimagination of bodily space can then also be seen as evidence for the more comprehensive turn towards early modernity in seventeenth-century China that some historians have written about.^83^ From this perspective, his innovations are not just responses to other developments, but active and powerful contributions in their own right to the wider transformations of society, culture, and thinking of which medicine formed a vital part.^84^ We have already outlined several other expressions of this transformation in the medical domain: the emergent professionalization of medical practice in cities like Suzhou; the critical reexamination of the textual sources that underpinned elite medicine that increasingly distanced the present from the past; and an emphasis on empirical observation rather than metaphysical speculation. We also saw how an interest in bodily flows in early modern economies resonates with concerns about the circulation of money and goods, and at least hinted at how this may be linked to anxieties and frustrations generated by societal change. One may even read Suzhou physicians’ preoccupation with the “bodily shell” as resonant of a broader process of individualization that is widely attested for this period and the new forms of boundary work it required. Other examples include efforts to understand the discrete functions of Chinese medicinals, efforts that mirror the formation of particularist ontologies in early modern Europe;^85^ a search for the meaning of individual lives that some commentators read into the philosophies of Wang Yangming 王陽明 (1472–1529) and his followers;^86^ the reemergence of portraiture as an important theme in late Ming painting, as well as a search for more individualist forms of artistic expression;^87^ and the cult of emotion, which required a new relationship to the individual self. Craig Clunas sums up these changes as a broad movement towards understanding people and things as autonomous entities rather than as embedded in distinctive social worlds.^88^

Such an interpretation of Ye Tianshi’s oeuvre – and of medical innovation in the Yangzi delta during the seventeenth century more generally – could make important contributions to our understanding of Chinese early modernity in relation to a more global early modern. Less ambitiously, but of no less significance, it orients the received history of Chinese medicine in new directions. For instance, Wang Qingren’s early nineteenth-century anatomical studies, introduced in the introduction to this essay, suddenly no longer appear as individualist aberrations within a tradition generally uninterested in mapping pathology onto concrete physical structures. Instead, they become one point on a longer trajectory of development in which Ye Tianshi was but another important instance. Likewise, the penetration of modern Western medicine into China may come to be seen not as demanding, finally, a turn towards the modern from a tradition hopelessly stuck in the intellectual cul-de-sac of correlative thinking, but as interrupting, perhaps fatally, a process of indigenous modernization already well underway.^89^

It should be clear from the above that I do not employ the term modernization in a teleological sense as implying convergence onto a singular endpoint.^90^ Rather, related by what one might term family resemblance, different instantiations of the modern are interesting to me because of the possibilities for translation they afford. Hence, while aligning Ye Tianshi’s imaginary of bodily space with contemporary anatomical knowledge and conceptions of disease has hopefully been a useful tool for communicating his ideas to an audience not versed in the Chinese medical literature, it remains just that – a tool. It is not an effort to constitute equivalence between these very different bodies, and even less so an attempt to measure how close or distant Ye Tianshi may have been from the “real body” described by biomedical anatomy. And yet, precisely because Ye Tianshi and biomedical anatomists engage with the same bodily materiality, it is also not surprising if, with due care and attention, it becomes possible to make one bodily imaginary intelligible to the other, and vice versa.

Enabling such dialogue surely should be one of the goals of a truly global history of medicine and science. To that end, I suggest we view Ye Tianshi’s body – comprised of structuring networks of flows – through the same sociohistorical lenses with which we view the various bodies that underpin contemporary biomedicine, namely as assemblages that succeed, at least momentarily, to align the multiple vectors that characterize a historical conjuncture into workable practices.^91^

I have only been able to sketch out the diverse problematics that, taken together, constituted the distinctive conjuncture that Ye Tianshi addressed in his clinical practice: the epidemiological crisis for which he conceived a workable and highly influential solution; his capacity to synthesize diverging strands of tradition into a strategic style of medical practice that was able to satisfy local patient demands without surrendering universal conceptions of bodily structure and function; and his ability, as a progressive physician from a non-literati background, to succeed in a highly competitive medical market, rising to the very top of a tradition in which continuity, orthodoxy, and descent remained important reference points.

It is beyond the scope of this paper to show in greater detail how precisely Ye Tianshi’s style of practice organized the problem space of medicine in early modern Suzhou. That it became the dominant style of prescribing throughout the Yangzi delta for almost two centuries attests to the success – or temporal stability – of his assemblage. I hope, however, to have at least made a case that any meaningful historical analysis must attend in detail to the materiality of the body that Ye Tianshi tried to grasp and manipulate to enable unhindered flow in the conduits and networks.

## Postscript

Between 1977 and 1980, a heated debate on the importance of the concept of *tong* for Chinese medicine erupted in the pages of the *Shandong Journal of Chinese Medicine*. The debate was sparked by an essay that proposed a novel solution to the Maoist project of creating a “new medicine” (*xinyi* 新醫) through a synthesis of Chinese and Western medical traditions.^92^ The key to fashioning this new medicine, the authors argued, could be found in the ancient Chinese notion of *tong* or “free flow.” They reasoned that because physiological functioning equates to the free flow of bodily processes, while pathology is its opposite, all medical practice must consequently be aimed at reconstituting free flow. They condensed their thinking into the slogan: “If there is disease there is no free flow; if there is free flow there is no disease” (*bing ze bu tong, tong ze bu bing* 病則不通, 通則不病). To those in the know, this catchphrase immediately evoked one of the most famous sayings in Chinese medicine often also cited by Ye Tianshi: “If there is no free flow, then there is pain. Therefore, unblocking removes the pain” (*bu tong ze tong, ze tong bu tong* 不通則痛, 則通不痛). Though lacking the seventeenth-century original’s more sophisticated homophonous pun, the new slogan succeeded in distilling the authors’ argument into a single memorable phrase that claimed universal validity, tied this knowledge firmly to the Chinese medical tradition, and resonated with the political sloganeering of the 1960s and 70s.

Not all the journal’s readers concurred with the proposed description of disease and flow, however. Physicians from all over Shandong as well as several neighboring provinces criticized the essay’s tendentious attempts to reduce a rich medical tradition to one single principle. There were supportive voices, too, that agreed with the essay’s goals as much as its main thesis.^93^ The stakes were high and the writing passionate. After all, nothing less than the very essence of Chinese medicine was being probed and the contours of a new world medicine were being defined. In the end, though, the debate fizzled out without noticeable impact on either Chinese or world medicine. Were it not for the digitalization of Chinese academic journals and the power of modern search engines, it is unlikely I would have picked up its traces after an interval of almost four decades.

And yet, this almost forgotten episode can tell us much about China, Chinese medicine, and Chinese science in the immediate aftermath of the Cultural Revolution (1966–76). Physicians in Shandong tentatively began playing with the possibility of letting a hundred flowers bloom once more, even as they tried to salvage something of value from the preceding “ten years of chaos.”^94^ Like Ye Tianshi, they placed the concept of *tong* at the center of their attempts to stabilize medical practice, albeit within a very different problem space from that of late imperial Suzhou. Even if some of its problematics would not have been readily intelligible to Ye Tianshi, these late twentieth-century attempts to understand the body in terms of free flow would have opened important channels for communication and translation between Ye and his Shandong colleagues.

The concept of *tong*, of course, refers not only to free flow but also to connectivity, continuity, and a reaching towards. Attending to the vectors that connect different problem spaces across time by way of shared problematics – and in the field of medicine, this specifically includes the materiality of the body – creates the possibility of dialogue and translation across traditions and sometimes apparently incommensurable worlds.

## Notes

1. Nathan Sivin, *Traditional Medicine in Contemporary China* (Ann Arbor, MI: Centre for Chinese Studies, The University of Michigan, 1987), p.14. Texts that articulate similar views include Shigehisa Kuriyama, *The Expressiveness of the Body and the Divergence of Greek and Chinese Medicine* (New York: Zone Books, 1999); Manfred Porkert, *The Theoretical Foundations of Chinese Medicine: Systems of Correspondence* (Cambridge, MA: MIT Press, 1974); Nathan Sivin, *Health Care in Eleventh-Century China* (New York: Springer, 2015); Paul U. Unschuld, *Medicine in China: A History of Ideas* (Berkeley, CA: University of California Press, 1985); and Paul U. Unschuld, *What is Medicine?: Western and Eastern Approaches to Healing* (Berkeley, CA: University of California Press, 2009).

2. Francesca Bray, *Technology and Gender: Fabrics of Power in Late Imperial China* (Berkeley, CA: University of California Press, 1997), p.303.

3. Sivin, *Traditional Medicine in Contemporary China*, p.14. For historical critique of conceptions of Chinese medicine as “holistic” see Volker Scheid, “Holism, Chinese Medicine and Systems Ideologies: Rewriting the Past to Imagine the Future,” in Angela Woods and Anne Whitehead (eds.) *The Edinburgh Companion to the Critical Medical Humanities* (Edinburgh: Edinburgh University Press, 2016), pp.66–86.

4. Benjamin A. Elman, *On Their Own Terms: Science in China, 1550–1900* (Cambridge, MA: Harvard University Press, 2005), p.227.

5. Elman, *On Their Own Terms: Science in China, 1550–1900*, pp.294–5. For an overview of Wang Qingren’s work and writings and their historical receptions see Bridie J. Andrews, “Wang Qingren and the History of Chinese Anatomy,” *Journal of Chinese Medicine* 36 (1991): 30–6.

6. Unschuld, *Medicine in China: A History of Ideas*, p.215.

7. Sean Hsiang-lin Lei, *Neither Donkey nor Horse: Medicine in the Struggle Over China’s Modernity* (Chicago, IL: University of Chicago Press, 2014), pp.82–6. All other “essential” characteristics of “traditional Chinese medicine” as we know them today, specifically “holism” (*zhengtiguan* 整體觀) and the practice of “pattern differentiation and disease determination” (*bianzheng lunzhi* 辨證論治), have equally modern origins. See Volker Scheid and Eric Karchmer, “History of Chinese Medicine, 1890–2010,” in Vincent Goosaert, Jan Kiely and John Lagerwey (eds.) *Modern Chinese Religion II: 1850–2015, Vol. 1* (Leiden: Brill, 2016): pp.141–96 for a review of these issues.

8. For a discussion of the composition and indication of these formulas as well as their use in contemporary clinical practice, see Volker Scheid, Andrew Ellis, Dan Bensky and Randol Barolet, *Chinese Herbal Medicine: Formulas and Strategies*, 2nd ed. (Seattle, WA: Eastland Press, 2009), pp.564–71.

9. I examine these transformations in Volker Scheid, “Transmitting Chinese Medicine: Changing Perceptions of Body, Pathology, and Treatment in Late Imperial China,” *East Asian Medicine: Tradition & Modernity* 8/2 (2015): 299–360.

10. T. J. Hinrichs and Linda L. Barnes, *Chinese Medicine and Healing: An Illustrated History* (Cambridge, MA: Harvard University Press, 2013).

11. Porkert, *The Theoretical Foundations of Chinese Medicine*; Kuriyama, *The Expressiveness of the Body*; Geoffrey E. R. Lloyd and Nathan Sivin, *The Way and the Word: Science and Medicine in Early China and Greece* (New Haven, CT: Yale University Press, 2002).

12. Unschuld, *Medicine in China: A History of Ideas*.

13. Sivin, *Traditional Medicine in Contemporary China*.

14. Yanhua Zhang, *Transforming Emotions with Chinese Medicine: An Ethnographic Account from Contemporary China* (Albany, NY: State University of New York Press, 2007).

15. Vivienne Lo, “The Influence of ‘Yangsheng’ culture on Early Chinese Medicine,” PhD thesis, School of Asian and African Studies (University of London) (1998). At the time, the acupuncture conduits were perceived as a series of distinctive conduits not united into one single system.

16. The two constituent parts of the *Inner Canon* are the *Basic Questions* (*Suwen* 素聞) and the *Spiritual Pivot* (*Lingshu* 黃靈樞). See Paul U. Unschuld, *Huang di nei jing su wen: Nature, Knowledge, Imagery in an Ancient Chinese Medical Text* (Berkeley, CA: University of California Press, 2003) for an exposition of the formation and content of the *Inner Canon*; and Paul U. Unschuld, Hermann Tessenow and Jinsheng Zheng, *Huang Di Nei Jing Su Wen: An Annotated Translation of Huang Di’s Inner Classic – Basic Questions*, 2 vols., (Berkeley, CA: University of California Press, 2011) and Paul U. Unschuld, *Huang di nei jing ling shu: The Ancient Classic on Needle Therapy*, 2nd ed. (Berkeley, CA: University of California Press, 2016) for translations of the two texts into English.

17. To avoid confusion, I capitalize organ names when the they refer to organ systems as imagined in Chinese medicine. Various conceptions of *tong* 通 can be found in *Basic Questions*, chapters 3, 5, and 10, and in *Spiritual Pivot*, chapter 10. Guo Aichun 郭靄春 (ed.), *Huangdi neijing suwen jiaozhu* 黃帝內經素問校註 (*The Inner Canon of Huangdi Basis Questions with Annotations*) (Beijing: Renmin Weisheng Chubanshe, 1989), pp.35–56, 72–106, 153–66; Guo Aichun 郭靄春 (ed.), *Huangdi neijing lingshu jiaozhu yuyi* 黃帝內經靈樞校註語譯 (*The Inner Canon of Huangdi Spiritual Pivot with Annotations and Commentary*) (Tianjin: Tianjin Kexue Jishu Chubanshe, 1989), pp.137–41.

18. Xu Zhicai徐之才, *Huangdi neijing lingshu jiashu yuyi* 黃帝內經靈樞校註語译譯 (*The Inner Canon of Huangdi Spiritual Pivot with Annotations and Translations*) (Hefei: Anhui Kexu Jichu Chubanshe, 1994).

19. Li Keguang 李克光 (ed.), *Jingui yaolüe* 金匮要略 (*Essentials from the Golden Casket*) (Beijing: Renmin Weisheng Chubanshe, 1989), pp.20–21.

20. The rewritings of Zhang Zhongjing’s biography are discussed by Miranda Brown, *The Art of Medicine in Early China: The Ancient and Medieval Origins of a Modern Archive* (New York: Cambridge University Press, 2015), pp.110–29. Asaf Moshe Goldschmidt, *The Evolution of Chinese Medicine : Song Dynasty, 960–1200* (London; New York: Routledge, 2009) examines the wider historical transformations that made Zhang Zhongjing’s works important for physicians from the eleventh century onward. Stephen Boyanton, “The Treatise on Cold Damage and the Formation of Literati Medicine: Social, Epidemiological, and Medical Change in China, 1000–1400,” PhD thesis, Graduate School of Arts and Sciences (2015) argues that Zhang Zhongjing’s central significance for the development of Chinese medicine predates the eleventh century. Marta E. Hanson, *Speaking of Epidemics in Chinese Medicine: Disease and the Geographic Imagination in Late Imperial China* (New York: Routledge, 2011), pp.124–5 shows that Zhang Zhongjing’s status was by no means undisputed, however, by later generations.

21. See, for instance, Li Jingwei 李經緯 and Deng Tietao 鄧鐵濤 (eds.), *Zhongyi dacidian* 中醫大辭典 (*Great Encyclopaedia of Chinese Medicine*) (Beijing: Renmin Weisheng Chubanshe, 1995), p.1324.

22. Figure 1 displays the different usage of the term *tong* 通 in the texts listed in Table 2 as a percentage of the total number of times that the term *tong* is listed as having been used by each individual author.

23. This usage appears to be particularly dominant amongst generalists writing after the Han and before the early Qing. It seems less important to authors in the cold damage (*shanghan* 傷寒) tradition, whose main concern remains focused on the *jing* 經 (conduits or divisions), which is not surprising given their central importance as locations of disease in cold damage disorders.

24. For biographies of Ye Tianshi and a synopsis of his work and influence on the development of Chinese medicine, see Huang Yingzhi 黃英志 (ed.), *Ye Tianshi yixue quanshu* 叶天士醫學全書 (*Complete Medical Works of Ye Tianshi*) (Beijing: Zhongguo Zhongyiyao Chubanshe, 1999), pp.1027–54 and Hanson, *Speaking of Epidemics in Chinese Medicine*, pp.115–18, 127–33, who also discusses his influence on the development of warmth disorder (*wenbing* 溫病) practice and Suzhou medicine.

25. The most important of these are *Case Records as a Guide to Clinical Patterns* (*Linzheng zhinan yi’an* 臨證指南醫案), a collection of case histories that provide access to Ye’s style of prescribing; the *Treatise on Warmth and Heat* (*Wenre lun* 溫熱論), a brief exposition of Ye’s methods for diagnosing and treating a distinctive type of febrile disorder; and *Systematic Differentiation of Warmth Disorders* (*Wenbing tiaobian* 溫病條辨), compiled by the scholar physician Wu Jutong 吴鞠通 (1758–1836) in an attempt to systematize Ye’s innovations in the treatment of febrile disorders.

26. Ye Tianshi 葉天士, “Linzheng zhinan yi’an pingben 臨證指南醫案評本 ([Xu Lingtai’s] Critical Version of the Case Records as Guides to Clinical Patterns),” in Wu Jiang 吳江 (ed): *Zengbu linzheng zhinan yi’an* 增補臨證指南醫案 (*Annotated Case Records as Guides to Clinical Patterns*) (Shanxi: Shanxi Kexue Jishu Chubanshe, 1999), pp.1–622.

27. This imaginary was shaped by the biographies in the *Draft of Qing History* (*Qingshi gao* 清史稿), by Suzhou physicians seeking to promote the image of their city, and by writers in the Jiangzi delta who opposed a southern medicine to that of the north. Hanson, *Speaking of Epidemics in Chinese Medicine*, discusses these processes in detail.

28. Zhai Zhongyuan 柴中元, *Wenbing qiuzhen* 溫病求真 (*Striving for Truth in Warmth Disorders*) (Beijing: Zhongguo zhongyiyao chubanshe, 1996) is an example of a contemporary author challenging received consensus.

29. Struggles over the importance of Ye Tianshi for the development of Chinese medicine reached a highpoint during the Republican period, when his innovations were widely criticized by modernizing physicians who emphasized cold damage therapeutics. See Eric Karchmer, “Ancient Formulas to Strengthen the Nation: Healing the Modern Chinese Body with the Treatise on Cold Damage,” *Asian Medicine: Tradition and Modernity* 8/2 (2013): 394–422.

30. For an example, see Elman, *On Their Own Terms: Science in China, 1550–1900*, p.236.

31. A vivid account of the impact of these transformations on Ming China between the mid-fourteenth and seventeenth centuries is provided by Timothy Brook, *The Confusions of Pleasure: Commerce and Culture in Ming China* (Berkeley, CA: University of California Press, 1998). The Prologue to Elman, *On Their Own Terms: Science in China, 1550–1900*, pp.3–23, summarizes these developments with reference to the history of science.

32. Volker Scheid, “Depression, Constraint, and the Liver: (Dis)assembling the Treatment of Emotion-Related Disorders in Chinese Medicine,” *Culture, Medicine and Psychiatry* 37, no. 1 (2013), pp.30–58.

33. Geng Song, *The Fragile Scholar: Power and Masculinity in Chinese Culture* (Hong Kong: Hong Kong University Press, 2004).

34. David Robinson, “Wu: The Arts of War,” in Craig Clunas and Jessica Harrison-Hall (eds.), *Ming: 50 Years That Changed China* (London: The British Museum, 2014), pp.112–55.

35. Paolo Santangelo, *Sentimental Education in Chinese History: An Interdisciplinary Textual Research on Ming and Qing Sources* (Leiden; Boston, MA: Brill, 2003); Paolo Santangelo, “Evaluations of Emotions in European and Chinese Traditions: Differences and Analogies,” *Monumenta Serica* 53 (2005): 401–27.

36. Elman, *On Their Own Terms: Science in China, 1550–1900*, pp.3–23.

37. Benjamin A. Elman, *From Philosophy to Philology: Intellectual and Social Aspects of Change in Late Imperial China*, 2nd rev. ed. (Los Angeles, CA: UCLA Asian Pacific Monograph Series, 2001); Benjamin A. Elman, “The Investigation of Things (Gewu 格物), Natural Studies (Gezhixue 格致學), and Evidental Studies (Kaozhengxue 考證學) in Late Imperial China, 1600–1800,” in Hans Ulrich Vogel and Günther Dux (eds.), *Concepts of Nature: A Chinese-European Cross-Cultural Perspective* (Leiden: Brill, 2010), pp.368–99.

38. For a first analysis of the wider impact of this movement in the medical domain see Benjamin A. Elman (ed.), *Antiquarianism, Language, and Medical Philology: From Early Modern to Modern Sino-Japanese Medical Discourses* (Leiden: Brill, 2015), and Elman, *On Their Own Terms: Science in China, 1550–1900*.

39. Hanson, *Speaking of Epidemics in Chinese Medicine*, p.92.

40. Angelika Messner, *Emotions in Late Imperial Chinese Medical Discourse: A Preliminary Report* (Naples: Ming Qing yanjiu, 2000), pp.197–215; Angelika Messner, “Emotions, Body, and Bodily Sensations within an Early Field of Expertise Knowledge in China,” in Paolo Santangelo and Ulrike Middendorf (eds.), *From Skin to Heart: Perceptions of Emotions and Bodily Sensations in Traditional Chinese Culture* (Wiesbaden: Otto Harrassowitz, 2006), pp.41–66.

41. For a detailed discussion, see Scheid, “Transmitting Chinese Medicine,” and also Hanson, *Speaking of Epidemics in Chinese Medicine*.

42. Use of the term “bodily shell” (*tike* 體殻), sometimes translated as “bodily husk,” in medical texts dates back at least to the tenth century. However, at the time it was used in depictions of bodily anatomy that focus on the internal organs and that were intended primarily for meditative practices. Rodo Pfister, “On the Meditative Use of the Body Maps Found in the Composite Text ‘Songs of the Bodily Husk’ (Ti ke ge),” *Curare* 39/1 (2016): 56–74. In the sixteenth century, it appears that *quke* 驅殻 was transformed into a term denoting the body’s external regions. In an essay that defines the meaning of different designation for the body and its parts, published in 1588, the influential author Gong Tingxian 龔廷賢, for instance, states: “The term shell implies an area. It is a collective term for a totality, like the area of a district.” (*Qu, qu ye. Shi zhong ming zhi dagang, ru qudi ye.* 軀, 區也; 是眾名之大總, 若區域也.) Gong Tingxian 龔廷賢, “Wan bing hui chun 萬病回春 (Restoration of Health from the Myriad Diseases),” in Li Shihua 李世華 and Wang Yuxue 王育學 (eds.), *Gongtingxian yixue quanshu (龔廷賢醫學全書)* (Beijing: Zhongguo zhongyiyao chubanshe, 1999, 1588), p.236.

43. For a detailed discussion, see Scheid, *Depression, Constraint, and the Liver*.

44. Xu Chunfu 徐春甫, *Systematic Great Compendium of Medicine Past and Present (古今醫統大全)* (Beijing: Kexue chubanshe, 1557), p.211.

45. John B. Henderson, *The Development and Decline of Chinese Cosmology* (New York: Columbia University Press, 1984).

46. Shigehisa Kuriyama, “The Historical Origins of *Katakori*,” *Japan Review* 9 (1997): 127–49.

47. Keiko Daidoji, “Treating Emotion-Related Disorders in Japanese Traditional Medicine: Language, Patients and Doctors’’, *Culture, Medicine and Psychiatry* 37 (2013): 59–80.

48. Ye Tianshi 葉天士, “Linzheng Zhinan Yi’an 臨證指南醫案 (A Compass of Clinical Patterns Based on Case Histories],” in Huang Yingzhi 黃英志 (ed.), *Ye Tianshi yixue quanshu* 葉天士醫學全書 (*The Complete Medical Works of Ye Tianshi*) (Beijing: Zhongguo zhongyiyao chubanshe, 1999, 1766), p.173.

49. Ye Tianshi, “Linzheng Zhinan Yi’an.” For the quantitative analysis, I have used the digital edition of this work contained in Shenzhou heshan kezhi kaifa youxian gongsi 深圳河山科技开发有限公司, “Zhongyi Gudai Jingdian 中醫古代典籍 (Classics of Chinese Medicine)” (2005).

50. Ye Tianshi, “Linzheng Zhinan Yi’an,” p.254.

51. Marta E. Hanson, “Robust Northerners and Delicate Southerners: The Nineteenth-Century Invention of a Southern Medical Tradition,” *Positions* 6/3 (1998): 515–50; Scheid, *Depression, Constraint, and the Liver*.

52. Ye Tianshi, “Linzheng Zhinan Yi’an,” p.95. Case of Mr Pu 浦, age 49.

53. Ye Tianshi, “Linzheng Zhinan Yi’an,” p. 242. Case of Mr Chen 陳.

54. For an introduction to four-sector diagnosis as represented in contemporary Chinese medical textbooks see Judith Farquhar, *Knowing Practice: The Clinical Encounter in Chinese Medicine* (Boulder, CO: Westview Press, 1994), pp.107–18.

55. Ye Tianshi, “Linzheng Zhinan Yi’an,” p.250. Synopsis by Shao Xinfu 邵新甫.

56. Porkert, *The Theoretical Foundations of Chinese Medicine*.

57. Donald John Harper, *Early Chinese Medical Literature: The Mawangdui Medical Manuscripts* (London: Kegan Paul International, 1998), p.82; Li Jianmin 李建民, *Si sheng zhi yu: Zhou Qin Han mai xue zhi yuan liu* 死生之域: 周秦漢脈血之源流 (*The Boundary of Life and Death: The Origin and Development of the Concepts of Vessels and Blood During the Zhou, Qin and Han*) (Taibei: Zhongyang yanjiuyuan lishi yuyan yanjiungsuo, 2000).

58. Harper, *Early Chinese Medical Literature*, p.83, argues that the etymology of the character *mai* 脈 itself provides evidence for the belief in such a network.

59. Unschuld, Tessenow and Zheng, *Huang Di Nei Jing Su Wen*, p.15.

60. Discussions of network pathologies can be found in *Basic Questions*, chapters 27, 28, 62, 63, and in *Spiritual Pivot*, chapters 17, 66. Aichun, *Huangdi neijing suwen jiaozhu*, pp.216–41, 242–61, 745–65, 766–85; Aichun, *Huangdi neijing lingshu jiaozhu yuyi*, pp.171–5, 434–41.

61. Ye Tianshi, “Linzheng zhinan yi’an,” p.295. Case of Mrs Chen 張.

62. Ye Tianshi, “Linzheng zhinan yi’an,” p.253. Case of Mr Long 龐.

63. Ye Tianshi, “Linzheng zhinan yi’an,” p.254. Synopsis by Hua Yutang 華玉堂.

64. Yu Chang 喻昌, “Yimen falü 醫門法律 (Precepts for Physicians),” in Chen Yi 陳熠 (ed.), *Yu Jiayan yixue quanshu* 喻嘉言醫學全書 (*Collected Medical Works of Yu Jiayan*) (Beijing: Zhongguo zhongyiyao chubanshe, 1643), pp.187–8.

65. Yu Chang, “Yimen falü 醫門法律 (Precepts for Physicians),” pp.187–8. Although this cannot be discussed here in detail, I believe that Yu Chang’s discussion of network structure and pathology prefigures Ye Tianshi’s later ideas in a much more profound manner than is acknowledged by historical and contemporary commentators.

66. In a discussion of a pediatric case that is followed by a long questions-and-answers session with his students, Yu Chang introduces the idea of the brain as the true ruler of the internal organs. In the history of Chinese medicine this is a radical innovation. Yu assimilates this concept, which suggest that he had access to Jesuit ideas, to Daoist internal alchemy practices, as well as to the new concept of the bodily shell. See Yu Chang 喻昌, “Yu yi cao 寓意草 (Notes on My Judgement),” in Chen Yi 陳熠 (ed.), *Yu Jiayan yixue quanshu* 喻嘉言醫學全書 (*Collected Medical Works of Yu Jiayan*) (Beijing: Zhongguo zhongyiyao chubanshe, 1999 [1643]), p.380.

67. This influence is clearly attested by Qian Qianyi in his foreword to Yu Chang’s “*Yimen falü* 醫門法律 (*Precepts for Physicians*).” See Qian Qianyi 錢謙益, “Yu Jiayan Yimen falü xu俞嘉言醫門法律序 (Foreword to Yu Jiayan’s Precepts for Physicians),” in Qian Cengjian 錢曾箋 (ed.), *Qian Muzhai quanji* 錢牧齋全集 (*Complete Edition of the Works of Qian Muzhai*) (Shanghai: Shanghai guji chubanshe, 2003), vol. 5, p.718.

68. Yuri Parfionovitch, Gyurme Dorje and Fernand Meyer (eds.), *Tibetan Medical Paintings: Illustrations to the Blue Beryl Treatise of Sangye Gyamtso (1653–1705)* (London: Serindia, 1992).

69. Qian Qianyi was treated by Yu Chang and wrote forewords to two of his books as discussed by Dai Zuming 戴祖銘, “Yu Chang and Qian Qianyi (Yu Chang yu Qian Qianyi 喻昌與錢謙益),” *Zhejiang Journal of Traditional Chinese Medicine* 浙江中醫雜誌 3 (2001): 152. Although there is no evidence that Fang Yizhi and Yu Chang met each other, Fang was an acquaintance of Qian Qianyi, with whom, he shared many common interests, including an interest in medicine. See Cheng Mengjun 陳孟君, “Yao bing weiyu de gingshen shi: Yi Qian Qianyi wei zhongxin kaocha 藥病為喻的精神史: 以錢謙益為中心的考察 (Metaphors of Disease and Medicine in the History of Thought: An Examination Focused on Qian Qianyi),” PhD thesis, Chinese Literature Department, National China University 國立中眾大學 (2014). I mention him here to give an intimation of the intellectual environment in which Yu Chang’s and Ye Tianshi’s innovations were generated.

70. Zhang Zhicong 張志聰, “Huangdi neijing lingshu jizhu 黃帝內靈樞集注,” in Zheng Lin 鄭林 (ed.), *Zhang Zhicong yixue quanshu* 張志聰醫學全書 (*Collected Medical Works by Zhang Zhicong*) (Beijing: Zhongguo zhongyiyao chubanshe, 1672), p.211.

71. Marta Hanson, “The Golden Mirror in the Imperial Court of the Qianlong Emperor, 1739–1742,” *Early Science and Medicine* 8/2 (2003): 137–8; Hanson, *Speaking of Epidemics in Chinese Medicine*, p.385.

72. Ye Tianshi, “Linzheng zhinan yi’an,” p.236. Case of Mr Zhu 朱.

73. Ye Tianshi, “Linzheng zhinan yi’an,” p.209. Case of Mr Cheng 程.

74. Ye Tianshi, “Linzheng zhinan yi’an,” p.139. Case of Mr Wang 王.

75. Ye Tianshi 葉天士, “Wenre Lun 溫熱論 (Treatise on Warmth and Heat),” in Huang Yingzhi 黃英志 (ed.), *Ye Tianshi Yixue Quanshu* 叶天士醫學全書 (*Complete Medical Works of Ye Tianshi*) (Beijing: Zhongguo zhongyiyao chubanshe, 1792), pp.339–46.

76. Ye Tianshi, “Linzheng zhinan yi’an,” p.180. Case of Mr Niu 牛.

77. Ye Tianshi, “Linzheng zhinan yi’an,” pp.243–4. Case of Mr Zhang 張.

78. Unschuld, Tessenow and Zheng, *Huang Di Nei Jing Su Wen*, p.18.

79. Ye Tianshi, *Wenre Lun*.

80. This statement is taken from the first case in the section on “All types of pain” (*Zhu tong* 諸痛). See Ye Tianshi, “Linzheng zhinan yi’an,” p.253. Case of Mr Chen 陳.

81. Ye Tianshi, “Linzheng zhinan yi’an,” p.222. Case of Mr Song 宋.

82. Ye Tianshi, “Linzheng zhinan yi’an,” p.253. Case of Mr Long 龐.

83. See, for instance, On-cho Ng, “The Epochal Concept of “Early Modernity” and the Intellectual History of Late Imperial China,” *Journal of World History* 14/1 (2003): 37–61, and Søren Clausen, “Early Modern China – a Preliminary Postmortem,” *Working Paper 84-00, Centre for Cultural Research, University of Aarhus* (2000).

84. See Chun-shu Chang and Shelley Hsueh-lun Chang, *Crisis and Transformation in Seventeenth-Century China: Society, Culture, and Modernity in Li Yü’s World* (Ann Arbor, MI: University of Michigan Press, 1992) for a comprehensive examination of modernization in early modern China. He Bian, “Assembling the Cure: Materia Medica and the Culture of Healing in Late Imperial China,” PhD thesis, Princeton University, Department of the History of Science (2014), argues that medicine was an intrinsic part of this transformation.

85. Bian, “Assembling the Cure,” p.14.

86. De Bary and William Theodore, *The Liberal Tradition in China* (Hong Kong; New York: Chinese University Press; Columbia University Press, 1983); De Bary and William Theodore, *Learning for One’s Self: Essays on the Individual in Neo-Confucian Thought* (New York: Columbia University Press, 1991).

87. Richard Ellis Vinograd, *Boundaries of the Self: Chinese Portraits, 1600–1900* (Cambridge, UK; New York: Cambridge University Press, 1992), pp.29–30.

88. Craig Clunas, “Artist and Subject in Ming Dynasty China,” *Proceedings of the British Academy* 105 (2000): 43–72.

89. Unschuld, *Medicine in China: A History of Ideas*, p.197, is the most influential advocate of such a position within the history of Chinese medicine. See also Paul U. Unschuld, *Medicine in China: A History of Pharmaceutics* (Berkeley, CA: University of California Press, 1986), pp.169–204.

90. This problematic has been widely discussed in debates about modernity and the global early modern, and among historians of science in the context of the famous Needham question. For overviews of these debates see Kenneth Pomeranz, “Areas, Networks, and the Search for ‘Early Modern’ East Asia,” in David L. Porter (ed.), *Comparative Early Modernities: 1100–1800* (London: Palgrave, 2012), pp.245–69 and other chapters in this volume, and Patrick K. O’Brian, “The Needham Question Updated: A Historiographical Survey and Elaboration,” *History of Technology* 29 (2009): 7–28.

91. On the multiple bodies biomedicine mobilizes in just one single location see Annemarie Mol, *The Body Multiple: Ontology in Medical Practice* (Durham: Duke University Press, 2002).

92. Shen Jie 申傑, Chen Kezhong 陳克忠, Shi Zhengchu 施政初 and Zhang Wanrong 張萬榮, “Shilun tongfa 試論通法」一文的看法: 從通與不通談起 (On the Unblocking Method: Starting from Flow and Blockage),” *Shandong Journal of Chinese Medicine* 山東醫藥 5 (1977): 8–10.

93. Shi Zhengchu 施政初, “Dui ‘Shilun Tongfa’ yi wen de kanfa 對’試論通法’一文的看法 (A View on ‘On the Unblocking Method’),” *Shandong Journal of Chinese Medicine* 山東醫藥 2 (1978), Editorial Committee of ‘Treatment Protocols’ for the Changwei District Chinese Medicine Association 昌濰地區中醫學會’治則’編寫組, “Yu ‘Shilun Tongfa’ de zuozhi shangque 與’試論通法’的作者商榷 (Debating the Author of ‘On the Unblocking Method’),” *Shandong Journal of Chinese Medicine* 山東醫藥 7 (1978): 51–3, Shen Jie 申傑, Chen Kezhong 陳克忠, Tai Shuren 太樹人, Yue Wenxian 岳文冼, Zhao Keshan 趙克山, Qi Xinglan 齊興蘭, Zhang Qiwen 張奇文, Xu Guoqian 徐國仟, Li Keshao 李克紹, Zhang Zhenyu 張珍玉, Ki Chengcai 劉承才, Shi Mushan 史慕山, Ren Chuanfang 莊傳芳 and 孫曾 Sun Zeng, “Shilun tongfa bitan 試論通法筆談 (Comments on ‘On the Unblocking Method’),” *Shandong Journal of Chinese Medicine* 山東醫藥 8 (1980): 50–6.

94. Roderick MacFarquhar and Michael Schoenhals, *Mao’s Last Revolution* (Cambridge, MA: Belknap Press of Harvard University Press, 2006); Anne F. Thurston, *Enemies of the People*, 1st ed. (New York: Knopf, 1987).

